# Diversity and Versatility in Small RNA-Mediated Regulation in Bacterial Pathogens

**DOI:** 10.3389/fmicb.2021.719977

**Published:** 2021-08-10

**Authors:** Brice Felden, Yoann Augagneur

**Affiliations:** Inserm, Bacterial Regulatory RNAs and Medicine (BRM) - UMR_S 1230, Rennes, France

**Keywords:** bacterial regulatory RNAs, noncoding RNAs, pathogens, small regulatory RNAs, small RNAs, sRNAs

## Abstract

Bacterial gene expression is under the control of a large set of molecules acting at multiple levels. In addition to the transcription factors (TFs) already known to be involved in global regulation of gene expression, small regulatory RNAs (sRNAs) are emerging as major players in gene regulatory networks, where they allow environmental adaptation and fitness. Developments in high-throughput screening have enabled their detection in the entire bacterial kingdom. These sRNAs influence a plethora of biological processes, including but not limited to outer membrane synthesis, metabolism, TF regulation, transcription termination, virulence, and antibiotic resistance and persistence. Almost always noncoding, they regulate target genes at the post-transcriptional level, usually through base-pair interactions with mRNAs, alone or with the help of dedicated chaperones. There is growing evidence that sRNA-mediated mechanisms of actions are far more diverse than initially thought, and that they go beyond the so-called *cis*- and *trans*-encoded classifications. These molecules can be derived and processed from 5' untranslated regions (UTRs), coding or non-coding sequences, and even from 3' UTRs. They usually act within the bacterial cytoplasm, but recent studies showed sRNAs in extracellular vesicles, where they influence host cell interactions. In this review, we highlight the various functions of sRNAs in bacterial pathogens, and focus on the increasing examples of widely diverse regulatory mechanisms that might compel us to reconsider what constitute the sRNA.

## Introduction: A Historical Overview of Regulatory RNA Discoveries

The central dogma of molecular biology states that DNA is replicated or transcribed into RNA, and that RNA is translated into proteins (Crick, 1958, 1970). This has contributed to the understanding (or perhaps widespread belief) that RNAs are just unstable intermediates (Pennisi, 2010). However, extensive research into these molecules has revealed that their purpose is not always to be translated. This has resulted in the identification of a new class called noncoding RNAs, which have gradually been shown to have a seemingly infinite range of biological functions and mechanisms of action (Cech and Steitz, 2014).

The first discoveries and characterizations of noncoding RNAs appeared in the late 1950s with the publication of studies on tRNA (Hoagland et al., 1958) and then rRNA (Cotter et al., 1967), both involved in protein translation. Various small noncoding RNAs (sRNAs) were subsequently identified in prokaryotes, with some being defined as regulatory RNAs due to their involvement in modulating bacterial metabolism (Wassarman et al., 1999). This was the case for 4.5S, transfer-messenger RNA (tmRNA), ribonuclease P (RNaseP) RNA, and 6S, all initially identified by fractionation in the late 1960s (Hindley, 1967). 4.5S is a 114-nucleotide (nt) component of the signal recognition particle (SRP) involved in protein secretion (Luirink and Dobberstein, 1994). It is essential, normally associates with ribosomes, and processed to be functional. tmRNA is a highly stable RNA that requires processing at the 5' and 3' ends by RNaseP and RNaseIII, respectively (Wassarman et al., 1999). It has sequential tRNA- and mRNA-like properties, and is involved in *trans*-translation for rescue of stalled ribosomes and protein degradation promotion (Janssen and Hayes, 2012). Initially identified as “10Sb” (Hindley, 1967), RNaseP is an essential RNA processed at the 3' end by RNaseE, and it forms a ribonucleoprotein complex with the RNaseP protein (Altman et al., 1989). RNaseP is responsible for the maturation of tRNA 5' termini, an essential step preceding the aminoacylation of mature tRNAs. *In vitro* studies on RNaseP revealed that the RNA moiety is the catalytic subunit (Guerrier-Takada et al., 1983), paving the way for the concept of ribozyme and later to that of the RNA world (Gilbert, 1986). One distinctive feature of RNaseP is that the RNA moiety recognizes a tRNA-like structure rather than Watson-Crick complexes, thus permitting the cleavage and maturation of 4.5S RNA or tmRNA or else the development of RNaseP-mediated RNA therapeutics through gene-selective mRNA cleavage (Augagneur et al., 2012; Kole et al., 2012; Derksen et al., 2015). The first non-rRNA, non-tRNA to be sequenced was 6S RNA from *Escherichia coli* (Brownlee, 1971), although it took almost 30 years for its role in sequestering the σ70 subunit of RNA polymerase to be demonstrated (Wassarman et al., 1999).

Meanwhile, other sRNAs including Spot42 and MicF were being discovered in *E. coli* (Ikemura and Dahlberg, 1973; Mizuno et al., 1984), and RNAI and RNAIII were soon thereafter discovered in the pathogen *Staphylococcus aureus* (Novick et al., 1989, 1993). As sRNAs were being discovered in virtually all bacteria, their regulatory mechanisms and biological functions began to be elucidated, bringing this class into the spotlight. Typically, sRNAs bind their target(s) (most often other RNAs by base-pairing and in a limited number of cases proteins) to modulate their expression at post-transcriptional level by influencing their stability and/or translation. However, it turns out that as a great variety of mechanisms was unraveled, a profusion of sRNA sub-categories emerged, and these regulatory RNAs began to be characterized as *cis*-encoded, *trans*-encoded, *cis*-acting, *trans*-acting, or even as sRNA-binding proteins. In eukaryotes, a similar abundance of non-mRNAs have been described. These include microRNAs, small interfering RNAs, Piwi-interacting RNAs, small noncoding RNAs, long noncoding RNAs, and circular RNAs, with these last ones acting as microRNA sponges in the cytoplasm (Chen et al., 2015; Burenina et al., 2017). It is therefore clear that the term “RNA” encompasses molecules that have a plethora of biological traits and mechanisms in both prokaryotes and eukaryotes (Cech and Steitz, 2014). In this review, we summarize, in a non-exhaustive manner, the current knowledge on sRNAs in bacterial pathogens, with a particular focus on *S. aureus*, *Listeria monocytogenes*, and *Salmonella*. We use specific examples to describe some usual and unusual features of this heterogenous group of transcripts whose precise categorization appears to be much more complicated than initially expected.

## sRNA Regulation is Required for a Variety of Biological Functions

Small regulatory RNAs are often described according to categories based on genome localization and their featured regulatory mechanisms. In this review, we take an opposite view, and start by discussing the diversity of biological functions in which sRNAs play a role. They are not constitutively expressed, but instead respond to environmental variations to modulate the gene expression of numerous targets (Wagner and Romby, 2015). These specific conditions include transition to the stationary phase, thermal shock, oxidative stress, and many other environmental challenges. Although, there are few sRNAs conserved over the bacterial kingdom (structural “housekeeping” sRNAs such as 6S, tmRNA, and RNaseP), they are often species- or order-specific. They have substantial advantages over transcription factors (TFs); they require less energy for production since translation initiation is unnecessary; they can act faster and reversibly; and they can still bind to multiple targets, which allow them to regulate a wide range of biological functions.

### sRNAs and Their Involvement in Virulence

In bacterial pathogens, it was an open question whether sRNAs were required for regulating virulence, either as activators or repressors. As sRNA molecular targets were being identified and regulatory functions in multiple pathways revealed, evidence has progressively emerged regarding their role in the direct or indirect control of virulence factors or the TFs that regulate virulence.

In *S. aureus*, the main sRNA involved in the commitment to virulence and quorum-sensing (QS) is RNAIII, an unusual 514-nt RNA that has an internal sequence coding for δ-hemolysin (Novick et al., 1993). RNAIII responds to cell density through the *agr* QS system and its TF, AgrA. The sRNA accumulates during bacterial growth, reaching a maximal concentration during the post-exponential phase (Singh and Ray, 2014). In doing this, it coordinates the transition from colonization to infection by directly or indirectly reprogramming the expression of a large set of genes (Bronesky et al., 2016; Table 1). Among the various virulence factors, RNAIII represses the expression of the TF Rot (Geisinger et al., 2006; Boisset et al., 2007), a repressor of numerous exotoxins that positively controls surface proteins such as Spa (Saïd-Salim et al., 2003; Oscarsson et al., 2006). By inhibiting this TF, RNAIII indirectly activates exotoxin production and inhibits surface protein expression. RNAIII and Rot inverted effects allow an effective switch between defense and offensive mode called Double Selector Switch (DSS; Nitzan et al., 2015). RNAIII also represses the expression of Spa, Coa, LytM, and Sbi by inhibiting ribosome binding or sometimes by promoting mRNA degradation (Table 1). Conversely, it upregulates the expression of α-hemolysin (Hla; α-toxin; Morfeldt et al., 1995) and MgrA TF, which inhibits surface protein expression (Luong et al., 2006; Gupta et al., 2015). We describe the actual mechanisms of actions against these targets later on in this review.

**Table 1:** Targets regulated by RNAIII.

Target	RNAIII effect	Encoded target function	Mechanism of action
*spa*	Repression	Adhesion and immune evasion	Translation inhibition and degradation by RNaseIII
*coa*		Adhesion	
*sa1000*		Adhesion	
*rot*		Transcription factor and toxin repressor	
*sbi*		Adhesion and immune evasion	Translation inhibition
*lytM*		Cell-wall metabolism and release of Spa	Translation inhibition
*hla*	Activation	Alpha toxin	Translation activator
*mgrA*		Transcription factor, inhibitor of surface proteins and autolysis, and activator of capsule synthesis	Stabilization of mRNA

Other *S. aureus*, sRNAs participate in virulence gene regulation, including SprD, the sRNA expressed from a pathogenicity island (Pichon and Felden, 2005). SprD downregulates the expression of Sbi, an immune evasion factor, at translational level (Chabelskaya et al., 2010). In the same study, the authors showed that the deletion of SprD resulted in the generation of a less-virulent strain in the mouse model of infection. This suggests that SprD controls the expression of other factors through yet-unknown mechanisms. Interestingly, Sbi is tightly controlled by both SprD and RNAIII, which are not expressed in *S. aureus* under the same conditions, and this suggests complementary roles for these two sRNAs in Sbi expression (Chabelskaya et al., 2014). Several other examples of sRNAs that positively or negatively control virulence exist in this bacterium, including SSR42, SprC, and RsaA (Morrison et al., 2012; Romilly et al., 2014; Le Pabic et al., 2015; Das et al., 2016).

In another low GC-content human pathogen *L. monocytogenes*, several sRNAs contribute to virulence (Toledo-Arana et al., 2007, 2009; Gripenland et al., 2010; Mellin and Cossart, 2012; Quereda and Cossart, 2017). First line of evidences came out with the deletions of blood-induced *rli38* and *rliB*, which resulting in attenuated or increased tissue colonization in a mouse model of infection, respectively. A study of *Listeria*’s intracellular transcriptome during growth in macrophages identified a large set of sRNAs, three of which (Rli31, Rli33-1, and Rli50) are directly associated with virulence (Mraheil et al., 2011). In addition, during intracellular growth, the blood-induced sRNA Rli27 upregulates the expression of Lmo0514, a cell-wall protein that has a pivotal role in virulence, as it is required for survival in plasma and for virulence in mice (Quereda et al., 2014, 2016).

In Gram-negative pathogens, most studies have focused on *Salmonella* Typhimurium and enterohemorrhagic *E. coli* (EHEC). In *Salmonella*, IsrM is a pathogenicity island-encoded sRNA with interesting features (Gong et al., 2011). Although, non-essential during growth *in vitro*, it is upregulated during infection, with high levels in the ileum. IsrM targets the SpoA and HilE mRNAs, which control the expression of *Salmonella* pathogenicity island 1 (SPI-1) genes. Deletion of *isrM* affects the bacterial invasion of epithelial cells, intracellular replication/survival in macrophages, and virulence in mice. In *E. coli* O157:H7 strain, several sRNAs are involved in virulence (Sauder and Kendall, 2018). Interestingly, the functions regulated by sRNAs differ in nonpathogenic *E. coli* and in EHEC, where core genome-encoded sRNAs can regulate virulence factors carried in pathogenicity islands. These include GlmY and GlmZ, sRNAs which control amino-sugar metabolism in nonpathogenic *E. coli* (Gruber and Sperandio, 2015) as well as being involved in type III secretion machinery in EHEC (Gruber and Sperandio, 2014). Similar to *S. aureus*, EHEC contains pathogenicity island-encoded sRNAs such as the antisense sRNAs Arl and sRNA350, both of which regulate bacterial virulence (Tobe et al., 2014; Bhatt et al., 2016). Additionally, some sRNAs such as DicF have both core genome-encoded and pathogenicity island-encoded copies (Melson and Kendall, 2019). When oxygen is limited, DicF binds *pchA* mRNAs, which encode a transcriptional activator of the type III secretion system. This allows access to the *pchA* ribosome binding site (RBS), promoting the expression of the activator and thereby increasing virulence (Melson and Kendall, 2019). Recent work on the sRNA, RyfA revealed its roles in virulence (mouse model) and survival (in human primary macrophages), both carried out by regulating genes coding for cell surface proteins and biofilm formation (Bessaiah et al., 2021). Other examples of sRNAs involved in virulence were reported in *Pseudomonas aeruginosa*, *Vibrio cholerae*, and *Helicobacter pylori*, and these have been thoroughly discussed in several articles and reviews (Pitman and Cho, 2015; Svensson and Sharma, 2016; Vannini et al., 2016; Ferrara et al., 2017; Quereda and Cossart, 2017; Zhao et al., 2018; Kinoshita-Daitoku et al., 2021).

### The Role of sRNAs in Host-Pathogen Interactions

In most of the examples discussed above, researchers began by identifying sRNAs and their targets in laboratory conditions or using *in silico* strategies before moving on to testing sRNA-deleted strains in animal models. These laboratory conditions only partially reproduce environmental cues, so some sRNA functions may be underestimated. Recently, the advent of deep-sequencing technologies filled in many blanks, enabling the study of sRNA expression and functions directly during host-pathogen interactions. Originally, the sRNA EsrF was predicted from transcriptomic data generated during EHEC infection of HeLa cells (Yang et al., 2015). EsrF senses high ammonium concentrations in the colon and promotes bacterial motility, host cell adhesion, and virulence in the colon (Jia et al., 2021). In *Salmonella*, dual RNA sequencing (RNA-seq) analysis revealed an activation of PinT during infection (Westermann et al., 2016). Similar to RNAIII, this sRNA plays an important role in chronological control of virulence factor expression in order to push the bacteria from the invasive to the virulent mode. PinT controls the SPI-1 and SPI-2 effectors required for intracellular survival, and causes pervasive changes in ~10% of the host’s coding and noncoding transcripts. A recent study using a novel MS2 affinity purification coupled with RNA sequencing (MAPS) technique (Lalaouna et al., 2017) in macrophages elegantly identified SteC, a novel PinT ligand that affects host actin rearrangement during infection (Correia Santos et al., 2021). The use of such new techniques should be extended to other pathogens, paving the way for the discovery of more sRNAs and new and better knowledge about their biological functions.

Another interesting thing about sRNAs is that their delivery into host cells from outer membrane vesicles allows them to modulate host-pathogen communications. While the characterization of sRNA content in extracellular vesicles is quite recent, links between sRNAs and host immune response were reported in *P. aeruginosa*, *H. pylori*, and *Vibrio fischeri* (Koeppen et al., 2016; Moriano-Gutierrez et al., 2020; Zhang et al., 2020). The RNA cargo of *S. aureus* was also recently characterized, revealing that the sRNAs RNAIII, RsaC, and SsrA (tmRNA) predominate. This suggests additional functions for these sRNAs in the control of immune host response (Joshi et al., 2020; Luz et al., 2021), as suggested in earlier reviews (Ellis and Kuehn, 2010; Avila-Calderón et al., 2015).

### sRNA-Mediated Antimicrobial Responses and Resistance

Although, the study of sRNAs and their roles in pathogenicity has inspired growing interest and uncovered new features, the biological functions of sRNAs go far beyond virulence. Besides being pathogenic, the emergence of bacterial strains resistant to antibiotic treatments is a serious public health issue, and several studies have therefore looked for correlations between sRNA expression and antibiotic challenges. These contributions to a better understanding of bacterial resistance and the role of sRNAs in these networks were recently reviewed (Felden and Cattoir, 2018).

In *Salmonella*, four sRNAs (sYJ5, SroA, sYJ75, and sYJ118) are upregulated when subjected to half the MIC of tigecycline (Yu and Schneiders, 2012), and the genetic deletion of *sroA* leads to reduced viability in the presence of that antibiotic. SroA exhibits the structural characteristics of a riboswitch, although its mechanism of action has not yet been characterized. In *E. coli*, RyhB is induced upon iron starvation, and it represses the expression of a large set of genes as well as participating in iron homeostasis (Massé and Gottesman, 2002; Chareyre and Mandin, 2018). During iron starvation, it is also involved in sensitivity to colicin Ia, an *E. coli*-specific bacteriocin produced to kill other *E. coli* strains (Salvail et al., 2013). To do this, RyhB binds *cirA* mRNAs, thereby activating its translation. CirA is a colicin Ia receptor and allows its translocation into the cell. Another study reported on the role of RyhB in antibiotic resistance after testing four classes of antibiotics (aminoglycosides, β-lactams, fluoroquinolones, and tetracycline; Chareyre et al., 2019). The authors showed that during iron starvation *ryhB* mutants were more susceptible to the aminoglycoside gentamicin as a result of the derepression of respiratory complexes Nuo and Sdh.

In *P. aeruginosa*, at least three sRNAs are known to be required for carbapenem resistance (Zhang et al., 2017; Sonnleitner et al., 2020). In the first study, Hi-GRIL-seq identified Sr0161 and ErsA as sRNA repressors of OprD, a porin involved in carbapenem antibiotic uptake. Their roles were functionally demonstrated in their respective deleted strains, in which meropenem susceptibility was significantly increased (Zhang et al., 2017). In the same article, Sr006 was shown to be involved in resistance to polymyxin B through the translation activation of PagL, an enzyme involved in lipopolysaccharide synthesis. In the second study, the sRNA CrcZ was shown to regulate carbapenem susceptibility through an indirect mechanism (Sonnleitner et al., 2020). By sequestering the Hfq protein when the preferred carbon source is exhausted, CrcZ prevents Hfq-mediated translational repression of OprP, another porin involved in carbapenem entry. Other studies have emphasized the role of sRNAs in *P. aeruginosa* and other Enterobacteriaceae, and are summarized in a recent review (Mediati et al., 2021).

In *S. aureus*, antibiotic exposure causes the specific expression of several sRNAs in the multidrug-resistant strain JKD6008 (Howden et al., 2013). Recently, the same authors identified a set of 18 sRNAs whose expressions vary under linezolid treatment (Gao et al., 2020). Although, no phenotypic variations were observed after genetic deletions of these sRNAs, which questions their actual roles in antibiotic resistance/adaptation, other studies have given direct evidence of direct sRNA involvement in antibiotic resistance. Depending on the strain, the sRNA SprX is encoded in one or more copies, and it inhibits translation of the SpoVG TF involved in glycopeptide and oxacillin resistance (Eyraud et al., 2014). The direct role of this sRNA was confirmed, as its deletion leads to moderately increased resistance, while its overexpression results in glycopeptide susceptibility. Antibiotic treatment failure is also largely attributed to the formation of persister cells, a subpopulation which is transiently tolerant of various antibiotic classes following entry into dormancy. A recent work identified the RNA antitoxin SprF1 in *S. aureus* as an RNA factor promoting persistence when challenged by ciprofloxacin and vancomycin at high doses (20x and 80x the MIC, respectively; Pinel-Marie et al., 2021). The authors demonstrated that this sRNA binds 70S ribosomes to slow translation and favor the entry into persistence, allowing survival until the antibiotic treatment is discontinued.

### sRNAs Span Many Other Functions

Studies of sRNAs in bacterial pathogens spotlight those involved in virulence or antibiotic resistance. However, many are involved in the regulation of other biological functions, especially fitness and adaptation. This is the case for instance for RsaE, RsaI, and RsaD in *S. aureus*. RsaE is the sRNA of about 100 nt which was identified by bioinformatics in intergenic regions (Geissmann et al., 2009), and which is conserved in Bacillales (Bohn et al., 2010). Transcriptomic and 2D-DIGE analysis of an *rsaE* mutant showed that this sRNA is involved in the regulation of several pathways connected to central metabolism, including the TCA cycle and metabolism of folate and malate. The first two targets to be characterized were the operon-encoded mRNAs *oppA* and *oppB* (Geissmann et al., 2009; Bohn et al., 2010). A subsequent search for molecular targets uncovered its role in arginine catabolism, with the arginase RocF downregulated when RsaE binds the mRNA (Rochat et al., 2018). In *Staphylococcus epidermidis*, a species which could become a threat due to spreading multidrug-resistant strains (Lee et al., 2018), RsaE participates in the regulation of the composition of the extracellular matrix (Schoenfelder et al., 2019). To do so, the sRNA undergoes processing which results in two forms regulating different targets (Lee et al., 2018). While the longer transcript interacts with *lrgA* mRNA (Rice et al., 2005) to cause both RNA decay and translational attenuation, the shorter species binds *icaR* mRNA (Cue et al., 2012) to inhibit translation. *Staphylococcus aureus* RsaI is the sRNA whose expression is tightly controlled by CcpA, a global regulator of carbon catabolite repression (Seidl et al., 2009; Bronesky et al., 2019). It is inhibited under high concentrations of glucose, and this is alleviated during the growth stationary phase (Geissmann et al., 2009). An in-depth characterization of the targetome using MAPS uncovered mRNA targets involved in sugar metabolism, glucose uptake, and biofilm formation, including mRNA transcription factors and remarkably, other sRNAs such as RsaE, RsaD, and RsaG (Bronesky et al., 2019). Among the target characterized, RsaI primirally acts as a post-transcriptional repressor. RsaD was discovered at the same time with other Rsa sRNAs (Geissmann et al., 2009). It might be part of the RsaI regulon, and is induced upon nitric oxide challenge (Bronesky et al., 2019). A study of the CodY regulon identified RsaD as a direct molecular target, and computational tools enabled the authors to find various RsaD mRNA targets including *alsS*, which encodes α-acetolactate synthase (Augagneur et al., 2020). Through post-transcriptional repression of *alsS*, RsaD redirects carbon overflow metabolism and regulates cell death during exposure to a weak acid stress.

In *Salmonella* Typhimurium, RydC was the first sRNA characterized as a regulator of membrane stability, binding *cfa* mRNAs and encoding cyclopropane fatty-acid (CFA) synthase (Fröhlich et al., 2013). Unlike most of the sRNAs discussed above, RydC upregulates CFA synthase by stabilization its mRNA. Other sRNAs such as RybB and MicA maintain envelope homeostasis (Papenfort et al., 2006), with RybB spurring the degradation of *omp* mRNAs upon activation of the envelope stress response, while MicA controls their decay.

SgrS is another sRNA that promotes the expression of some of its targets (Papenfort et al., 2013). This sRNA is involved in glucose homeostasis through the activation and repression of several targets (Vanderpool and Gottesman, 2004). SgrS was first identified as a repressor of the phosphotransferase system (PTS), preventing the translocation of sugars into cells when the intracellular concentration of phosphorylated sugars is too high (Vanderpool and Gottesman, 2004). The sRNA also activates the translation of YigL, a phosphatase involved in detoxification of phosphosugars, thereby allowing diffusion of dephosphorylated sugars outside the cell membrane (Papenfort et al., 2013). While GlmY and GlmZ are both involved in virulence in EHEC (see section above on sRNAs and Their Involvement in Virulence), they also have other conserved regulatory activities in nonpathogenic *E. coli*, including amino sugar metabolism and cell envelope synthesis (Urban and Vogel, 2008; Göpel et al., 2014; Sauder and Kendall, 2018).

Initially identified in nonpathogenic *E. coli*, RyhB is involved in iron homeostasis, a critical factor in cellular processes (Massé and Gottesman, 2002). It is under the control of Fur, the TF that represses iron acquisition genes and RyhB when iron is abundant. RyhB downregulates iron-storing and iron-using proteins and these are therefore indirectly activated when Fur represses RyhB. In *Salmonella*, two paralogs have been identified: RyhB-1 and RyhB-2 (Kim, 2016). These share a 33-bp sequence with perfect identity, and can thus regulate the same targets (Kim and Kwon, 2013). Although their promoters are recognized by Fur, their expression profiles vary (Padalon-Brauch et al., 2008): *ryhB-1* is induced under iron-depleted conditions or oxidative stress, while maximal *ryhB-2* expression is seen during the stationary phase. Additional roles for RyhB paralogs were reported in *Salmonella* (Kim, 2016). These include involvement in nitrate homeostasis (Teixidó et al., 2010), oxidative stress, intracellular survival in macrophages, and control of SPI-1 and Type III secretion system gene expression (Leclerc et al., 2013; Calderón et al., 2014; Peñaloza et al., 2021).

DapZ is an 80-nt sRNA identified in *Salmonella* and transcribed from the 3' region of *dapB* (Chao et al., 2012). It is involved in the uptake of nutrient and signaling molecules. *Via* base-pairing, DapZ modulates the synthesis of ABC transporters Opp and Dpp, which encode oligopeptide and dipeptide permeases, respectively.

The ensemble of sRNA studies highlights their roles in many biological functions ranging from virulence to antibiotic resistance, and even including the regulation of TF expression and transcription termination. While sRNA research involving bacterial pathogens has often concentrated on virulence or antibiotic resistance because of public health issues, they are actually involved in all aspects of bacterial biology. There are about as many sRNAs discovered as TFs factors inventoried, which indicates that sRNAs are among the key players in transcriptional and post-transcriptional regulation of gene expression.

Just as there has been a large number of biological functions identified, the functional categorization of their mechanisms of production or regulation is broad and diverse, with new features constantly unraveled. sRNAs are a heterogeneous group of transcripts with lengths usually ranging from 50 to 500 nucleotides. They are usually highly structured, have greater stability than mRNAs, and use their base-pairing abilities to interact with and regulate their targets. To promote interaction with a target, a chaperone is sometimes necessary (Kavita et al., 2018). Based on their features, they can be categorized in multiple manners. In the following sections, we will first present the canonical features and mechanisms of action, then use selected examples to illustrate original features to showcase the diversity and versatility uncovered at the same time as the discovery of sRNAs exploded, suggesting that more surprises are in store.

## sRNAs That Interact with Proteins

While most sRNAs use base-pairing to activate or repress the expression of their targets, some bind proteins to form ribonucleoprotein complexes (Pichon and Felden, 2007). These complexes are involved in the metabolism of DNA (regulation of plasmids and DNA transfers), RNAs, and proteins. When it comes to RNA metabolism, sRNAs can be involved in transcription through 6S sRNAs, in RNA maturation *via* RNAseP, or subject to decay when recognized by endo- or exo-ribonucleases (Wassarman et al., 1999; Hausmann et al., 2017; Redder, 2018; Le Scornet and Redder, 2019). In addition, some are assisted by *trans*-acting chaperones to facilitate recognition of “bait” and “prey” (Pichon and Felden, 2007). At protein level, they are important for global translation machinery and quality control (including tmRNAs involved in ribosome rescue), for protein trafficking with SRP and 4.5S RNA, and for sequestration of global regulators. Most of these characteristics are beyond the scope of this review, and we will just discuss the need for protein chaperones to stabilize interactions as well as the role of sRNAs in protein sequestration, both usually found in Gram-negative bacteria.

### The Role of Protein Chaperones in Stabilizing RNA–RNA Interactions

**Figure 1:**
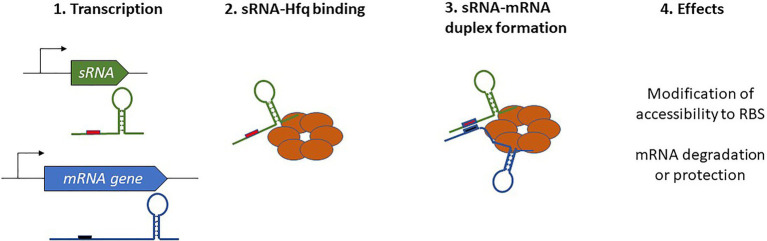
Schematic overview of Hfq-dependent small regulatory RNA (sRNA) complex formation and the resulting effects on target mRNAs.

In Gram-negative bacteria, the activity of numerous sRNAs relies on them acting in concert with a RNA chaperone protein. The most prevalent of these is Hfq, often referred to as the “RNA matchmaker” (Updegrove et al., 2016). Hfq is a homohexameric ring-shaped protein with at least three different RNA-binding domains shared between the rim and the proximal and distal faces of the hexamer (Gorski et al., 2017; Carrier et al., 2018). The proximal face specifically recognizes U-rich tracts, often associated with *rho*-independent transcription terminators, whereas the distal face anneals sRNAs through A-rich regions. Similar to the proximal face, the rim of the hexamer also binds U-rich regions. Not only does the binding with Hfq stabilize the sRNA and protect it from RNases, but also its ability to bind simultaneously with mRNAs and favors the formation of mRNA-sRNA duplexes. Indeed, this feature allows for the appropriate presentation of the sRNA seed region to an mRNA target, which in turn affects mRNA stability or translation (Figure 1). With the help of Hfq, only a short and conserved sRNA seed sequence is necessary for annealing with the target and promotion of regulatory activity. For instance, just seven nucleotides are responsible for the degradation of *omp* mRNAs by RybB (Balbontín et al., 2010; Papenfort et al., 2010). In addition to its RNA chaperone activity, Hfq can also directly influence translation by binding the 5' UTR of *cirA* mRNAs as well as participate in RNaseE recruitment to induce the rapid degradation of target mRNAs (Ikeda et al., 2011; Salvail et al., 2013). This indicates a novel role and mechanisms of action for this sRNA, and these are extensively discussed in a recent review by Ng Kwan Lim et al. (2021).

Whether Hfq was the sole RNA chaperone, which was answered using a Grad-seq, technology first used in *Salmonella* (Smirnov et al., 2016). This enabled the discovery of several sRNAs such as RaiZ, associated with ProQ RNA-binding protein. The authors demonstrated that RaiZ represses the expression of the histone-like protein HU-α and that ProQ acts as a chaperone in RaiZ transcription stabilization rather than facilitating base-pairing. RIL-seq then enabled a redefinition of the interactomes of Hfq and ProQ, revealing their overlapping and competing roles (Melamed et al., 2020). In other Gram-negative bacteria such as *Legionella* and meningococcus, identification and exploration of ProQ and ProQ-like proteins has demonstrated that Hfq is not the only player in the RNA-binding hub (Attaiech et al., 2016; Bauriedl et al., 2020).

Conversely, the need for the RNA chaperone to enhance sRNA-mRNA interactions seems marginal in Gram-positive bacteria, as no Hfq homologs exist in streptococci or lactobacilli (Mellin and Cossart, 2012). Investigations into Hfq functions in *S. aureus* and *Bacillus subtilis* using deleted strains did not result in any significant phenotypes, and several Hfq-independent sRNAs have now been described (Bohn et al., 2007; Rochat et al., 2015). However, studies in *L. monocytogenes* did reveal a functional albeit sometimes minor role for Hfq in sRNA-mediated regulation or tolerance to stress and virulence (Christiansen et al., 2004; Mandin et al., 2007). Co-immunoprecipitation coupled to enzymatic RNA sequencing produced the first evidence of interaction between Hfq and three sRNAs (Christiansen et al., 2006). Among these, LhtA regulates the translation and degradation of its mRNA targets through an Hfq-dependent antisense mechanism (Nielsen et al., 2010, 2011). In contrast, the multicopy sRNA LhrC identified in the same screen (Christiansen et al., 2006) does not require Hfq to stabilize its interaction with *lapB*, its target mRNA (Sievers et al., 2014). Together, these results indicate a controversial and probably dispensable role for Hfq in post-transcriptional control of gene expression in several Gram-positive species. This points to major differences in sRNA-mediated regulation between Gram-negative and Gram-positive bacteria, or perhaps to the existence of other RNA chaperones (Jørgensen et al., 2020) yet to be discovered.

### Sequestration of Proteins by sRNAs

Small regulatory RNAs do not necessarily anneal to the RNA chaperone to enhance target recognition. They can bind proteins to inhibit their action by mimicking the structures of their target mRNAs, although examples of this are limited, and mostly restricted to Gram-negative bacteria. For instance, the homodimeric RNA-binding protein CsrA is sequestered by CsrB and CsrC sRNAs, which antagonize its activity (Liu et al., 1997; Weilbacher et al., 2003). This CsrA carbon storage regulator normally represses gene expression by direct binding with the translation initiation region of its mRNA targets, and only a few examples of gene activation have been reported (Pourciau et al., 2020). This sRNA is associated with post-transcriptional control of its targets rather than the transcriptional control usually described for TFs, and it affects translation initiation, RNA stabilization, and transcription termination. For optimal sequestration of the protein, CsrB and CsrC sRNAs contain multiple CsrA-binding sites, with ~18 in CsrB. That sRNA harbors repeated sequence elements, including a GGA motif present in the loop of hairpin structures or in single-stranded regions (Romeo, 1998). These GGA seeds decoy the CsrA target motifs usually present near the SD sequence of target sRNAs. Similar decoy seed regions are found in several CsrB homologs in various bacterial species (Babitzke and Romeo, 2007; Janssen et al., 2018; Müller et al., 2019; Sonnleitner et al., 2020). sRNAs in the CsrB family have an impact on the CsrA regulon and on various physiological functions such as biofilm formation, host-microbe interaction, and virulence (Vakulskas et al., 2015). Their expression relies on the BarA/UvrY two-component system, increasing under nutrient limitation or cellular stress, and decreasing through RNA degradation in the presence of the preferred carbon source (Pourciau et al., 2020). In addition, a reciprocal regulation was also revealed, as free CsrA is mandatory for CsrB/C synthesis. Unusually, sRNAs in the Csr family have short half-lives, allowing for the rapid adjustment of CsrA activity in response to environmental cues. Ultimately, it has been shown that CrsB homologs do not necessarily sequester CsrA. In *P. aeruginosa*, CsrZ antagonizes Hfq, resulting in differential carbapenem susceptibility during growth on different carbon sources (Sonnleitner et al., 2020). More roles were reported for CsrA in *B. subtilis*, where it allows the formation of complexes between the sRNA and its mRNA target, therefore, working as an Hfq-like or ProQ-like RNA chaperone (Müller et al., 2019). Additionally, some sRNAs belong to type III toxin-antitoxin (TA) systems in which the regulatory RNA binds and sequesters its cognate toxin (Brantl and Jahn, 2015).

## Classification of Base-Pairing Acting sRNAs According to Their Origins and Mechanisms of Action

Base-pairing sRNAs are expressed from a large variety of loci, and they can be generated from the genome or plasmids (Tomizawa et al., 1981). Within the genome, they are transcribed from both the core genome and accessory genomes such as pathogenicity islands (Pichon and Felden, 2005), and either antisense to their target or *trans*-encoded from a locus distant to their cognate targets.

### Antisense-Encoded sRNAs and Their Mechanisms of Action

**Figure 2:**
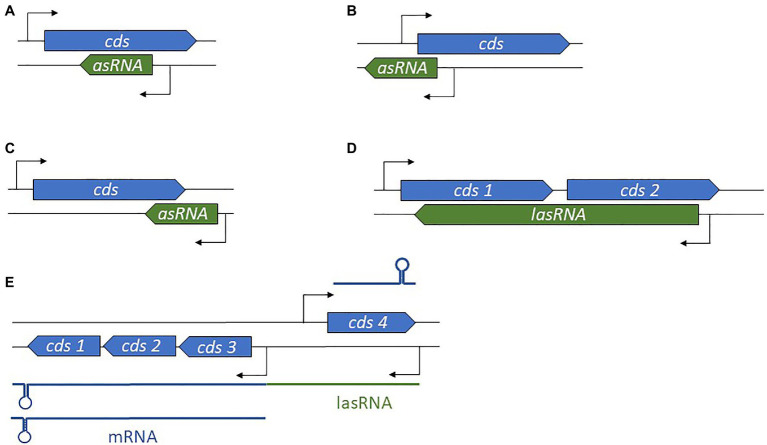
Diversity in antisense RNA (asRNA) genomic locations and lengths. **(A)** Position of an intragenic asRNA. **(B)** An asRNA complementary to and overlapping the 5' untranslated region (UTR). **(C)** An asRNA complementary to and overlapping the 3' UTR. **(D)** A long asRNA (lasRNA) covers multiple genes. **(E)** In the concept of the excludon, an lasRNA controls the expression of an mRNA expressed from the opposite strand, and also encodes mRNA. *cds*, coding sequence.

**Figure 3:**
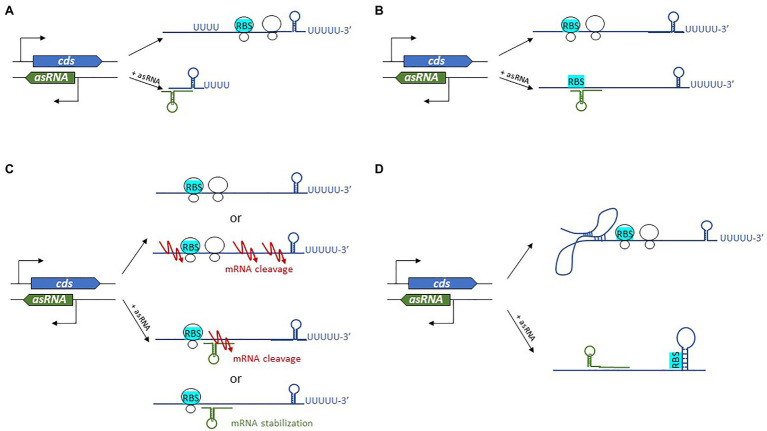
Diverse regulatory mechanisms are employed by *cis*-encoded antisense RNAs (asRNAs). **(A)** Premature transcription termination results in transcription attenuation. **(B)** When ribosomal loading is blocked, translation is inhibited. **(C)** Modification of mRNA decay after mRNA stabilization or RNase recruitment. **(D)** RNA pseudoknot formation leads to modified RBS accessibility. *cds*, coding sequence and RBS, ribosome binding site.

Antisense-encoded sRNAs (asRNAs), also called *cis*-encoded antisense RNAs, are encoded on the DNA strand opposite to their mRNA target. They share extensive and perfect complementary with part of their mRNA target, allowing efficient regulation without usually requiring the RNA chaperone. They were originally discovered in plasmids and other genetic mobile elements such as phages and transposons (Tomizawa et al., 1981; Brantl, 2002), but we now know that they are present throughout in the genome. For some asRNAs, additional targets transcribed from distant loci have been reported, indicating that they can also act as *trans*-encoded sRNAs. asRNAs have a large range of lengths, from ~50 to several thousand nucleotides. They can be transcribed from a DNA strand whose coordinates are within the coding region (CDS) of the opposite strand, overlapping with a CDS (at the 5' or 3' UTR), or in the case of the very long RNAs (lasRNAs), antisense to a complete operon (Dühring et al., 2006; Figures 2A–D). Their identification is challenging, as wide antisense transcription was reported in several pathogens including *S. aureus*, making it difficult to distinguish between “true” asRNAs (having their own promoters) and transcriptional noise (Lasa et al., 2011). Of the thousands of asRNAs identified in a single species, only a tiny subset has been functionally characterized. Plasmid-encoded asRNAs are often constitutively expressed and involved in specific biological functions such as plasmid replication, conjugation, post-segregational killing, and transposition (Wagner et al., 2002). Conversely, genome-encoded asRNAs are primarily linked to the functions of the protein encoded from the opposite strand, and their expression is modulated under specific conditions (Brantl, 2007). Most often, asRNAs are responsible for post-transcriptional regulation and they exhibit a large array of mechanisms of actions that include transcription attenuation or inhibition, modification of mRNA decay, RNA pseudoknot formation, and primer maturation inhibition (Brantl, 2007; Figure 3). In a few cases, activating mechanisms have been reported. Similar mechanisms of action have also been described for *trans*-encoded sRNAs. We will not discuss the inhibition of primer maturation here, as this mechanism of action is mostly restricted to plasmid replication and is not generally applicable to most asRNAs or *trans*-encoded sRNAs.

#### Transcription Attenuation

Transcription attenuation occurs when a termination structure is formed in the target mRNA after asRNA annealing (Figure 3A). This was first observed as a means to control copy numbers in staphylococcal and streptococcal plasmids (Novick et al., 1989; Brantl et al., 1993; Brantl, 2002). In these bacteria, *rep* mRNAs can adopt two mutually exclusive conformations depending on the presence or absence of the asRNA. Expression of the asRNA induces a terminating stem-loop that causes the premature termination of *rep* transcription upstream from the RBS, thereby preventing translation initiation. Conversely, in the absence of the asRNA, the natural conformation of *rep* mRNA prevents stem-loops from forming. This structure enables the transcription of full-length *rep* mRNA, permitting translation and therefore plasmid replication. Such a mechanism of action has been reported in the post-transcriptional control of the virulence gene *icsA* in *Shigella flexneri* (Giangrossi et al., 2010), as well as in iron transport in the fish pathogen *Vibrio anguillarum* (Stork et al., 2007). That first study identified RnaG, an asRNA expressed from the opposite strand of *iscA*. Upon RnaG expression, the formation of a heteroduplex induces the formation of an intrinsic terminator, thus leads to transcription termination (Giangrossi et al., 2010). In the second study, the expression of the asRNA RNAβ enabled transcription termination within the *fatDCBA-angRT* transport and siderophore biosynthesis operon, resulting in approximately 17-fold higher expression levels of *fatDCBA* genes than *angRT* ones (Stork et al., 2007).

#### Translation Inhibition

Translation inhibition is probably the most well-known RNA-mediated mechanism employed to post-transcriptionally regulate gene expression (Figure 3B). Here, the asRNA presents perfect complementarity with the RBS of the mRNA transcribed from the opposite strand, so it competes with ribosome loading. Several examples have been described in plasmidic asRNAs, such as RNAII which controls plasmid pLS1 replication by binding the *repB* RBS (Brantl, 2007). A similar mechanism was reported in FinP, an asRNA which represses the expression of TraJ by blocking ribosomal access to *traJ* mRNA, inhibiting conjugation. For optimal regulation in this case, the FinO RNA chaperone is necessary to facilitate RNA–RNA duplex formation and to protect FinP against RNaseE-mediated RNA degradation (Arthur et al., 2003).

#### Modification of mRNA Decay

**Figure 4:**
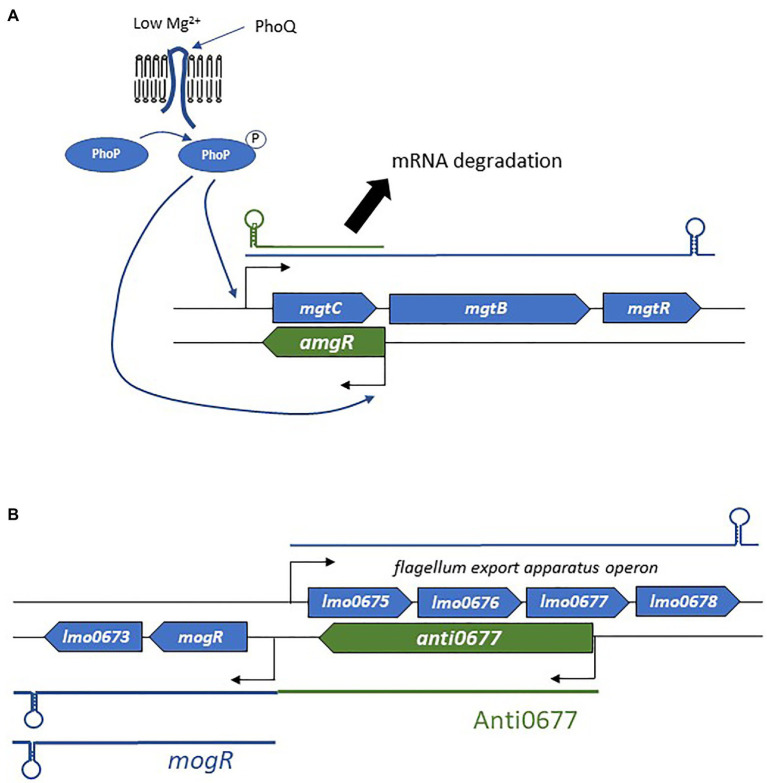
Examples of the mechanism of actions employed by asRNAs. **(A)** Regulation of the *mgtCBR* operon and the role of AmgR in degradation of *mgtC* mRNA. **(B)** Schematic representation of the flagellum biosynthesis excludon downregulated by the long asRNA Anti0677.

The binding of the asRNA to its complementary mRNA target also modifies mRNA decay (Brantl, 2007; Figure 3C). Most often, repression is observed through endoribonuclease cleavage of RNA. Indeed, double-stranded RNAs and the asRNA-mRNA heteroduplex are substrates of RNase III. This enzyme preferentially recognizes long dsRNA segments, which are then cleaved. In *S. aureus*, genome-wide antisense transcription activity was shown to be coupled with RNase III processing of mRNA/asRNA duplexes (Lasa et al., 2011). Such a mechanism may be involved in the post-transcriptional modulation of mRNA counts, as this duplex formation causes at least 75% of the mRNAs to be specifically cleaved by RNase III. In the same study, the authors reported similar trends in *B. subtilis*, *Enterococcus faecalis*, and *L. monocytogenes*. RNase E is another RNase that participates in mRNA decay. A good example is that of the *Salmonella* asRNA AmgR, expressed from the opposite strand of *mgtC* (Lee and Groisman, 2010; Figure 4A). During phagocytosis, the PhoPQ two-component system senses low concentrations of Mg^2+^, leading to signal transduction and the induction of the expression of several genes including the *mgtCBR* operon (Alix and Blanc-Potard, 2008). In addition, PhoP activates the expression of AmgR, which binds *mgtC* mRNAs, and favors its degradation *via* RNase E but not RNase III. In the absence of AmgR, the operon is transcribed, promoting bacterial virulence (Lee and Groisman, 2010). Although, the preferred mode of action is the promotion of RNA degradation, there have also been reports of inhibition of mRNA degradation and therefore of positive post-transcriptional effects of asRNAs (Sesto et al., 2013; Svensson and Sharma, 2016).

In bacterial pathogens, several examples of asRNAs overlapping an RBS have been described, and they go beyond plasmid replication-related functions (Thomason and Storz, 2010; Lejars and Hajnsdorf, 2020). This is the case in *Pseudomonas*, *Salmonella*, *Shigella*, *Clostridioides difficile*, *L. monocytogenes*, and *S. aureus*, although precise demonstrations of this mechanism of action are lacking, and it is often linked to transcript abundance variations, as recently reviewed (Lejars and Hajnsdorf, 2020). This indicates that translation inhibition and mRNA decay are often concomitant, as we will see for RNAIII in the next section.

#### RNA Pseudoknot Formation

RNA pseudoknot formation can be altered by the presence of asRNAs (Asano and Mizobuchi, 1998a,b). In this case, conformational changes are responsible for regulation as well as for transcription attenuation (Figure 3D), with the translation of *repZ* dependent on the formation of a pseudoknot upstream of the RBS. Binding of the asRNA Inc. to *repZ* mRNA blocks the formation of this pseudoknot and thus prevents translation (Brantl, 2007).

#### The Excludon

In addition to the canonical localizations and mechanisms of action, a novel concept in bacterial asRNA-mediated gene regulation has also emerged: the excludon (Sesto et al., 2013; Toledo-Arana and Lasa, 2020). This was first delineated in *L. monocytogenes* soon after the identification of a group of lasRNAs (Wurtzel et al., 2012). In an excludon, transcription of lasRNAs is initiated from a promoter located on the opposite strand of the CDS (Figure 2E). One interesting feature of the excludon is that transcription continues beyond the overlapping region, also encompassing the coding regions located downstream from the actual asRNA locus. Through this atypical mode of biogenesis, the transcript possesses dual functions, with the 5' antisense regulator of the gene(s) expressed from the opposite strand, while the 3' moiety encodes one or more proteins. Several excludons are reported in *L. monocytogenes*, and the functions encoded within the locus (i.e., lasRNA and mRNA) are closely related (Sesto et al., 2013). In that pathogen, excludons were shown (or predicted) to be involved in flagellum biosynthesis, a permease-efflux pump, or utilization of carbon sources. As these examples have already been well-described (Sesto et al., 2013), we will just discuss the mechanism of action employed in the biosynthesis of flagella (Figure 4B), extracellular appendages important for cell motility, biofilm formation, and host cell invasion (O’Neil and Marquis, 2006; Lemon et al., 2007). The excludon is composed of four genes expressed from the positive strand that encodes the flagellum export apparatus, *lmo0675*, *lmo0676*, *lmo0677*, and *lmo0678*. Two genes of this operon are encoded divergently, including the *mogR* transcriptional regulator of the flagellum export apparatus (Figure 4B). The lasRNA Anti0677 is expressed from a σ^B^-dependent promoter far upstream from *mogR* and as an antisense of *lmo0677*. When bacteria are not subjected to stress, the flagellum export apparatus operon is under the sole control of the transcriptional repressor MogR. Under stress conditions, σ^B^ promotes transcription of Anti0677, leading to an efficient switch-off of the flagellum production through direct antisense inhibition of the operon and increased MogR expression in both *mogR* mRNAs and Anti0667 lasRNAs. Recently, a similar “noncontiguous operon” organization leading to transcriptional interference coupled with endoribonuclease-mediated cleavage was reported in *S. aureus* (Sáenz-Lahoya et al., 2019).

#### sRNAs in Type I Toxin-Antitoxin Systems

Several asRNAs belong to type I TA systems. Type I TA modules are composed of a stable toxic peptide and a labile asRNA that inhibits toxin expression, although the existence of divergently expressed genes leading to the expression of a *trans*-encoded sRNA has also been reported (Brantl and Jahn, 2015). Initially discovered on plasmids, where their role in post-segregational killing prevents plasmid loss during cell division, overexpression of these systems induces small membrane-damaging peptides, which leads to cell death, global translation inhibition, and commitment to persistence. Their mechanisms of action often rely on a perfect complementarity between the RNA antitoxin and the mRNA target, and are associated with translation inhibition and dsRNA-degradation of the heteroduplex through RNase III recruitment (Wen and Fozo, 2014). However, several new features have also been reported, particularly in *S. aureus*.

The type I TA SprA/SprA_AS_ system identified in a pathogenicity island (Pichon and Felden, 2005), exemplified the fact that an asRNA regulates in *trans* the translation of its cognate antitoxin (Sayed et al., 2012). Here, the two genes overlap at their 3' ends, suggesting that the putative mechanism of action is not the annealing of the RNA antitoxin onto the RBS. Structural probing of SprA1 RNAs showed the presence of two pseudoknots and a 5' stem-loop that unfavored RBS accessibility. Upon SprA1_AS_ binding, an internal RNA pseudoknot of SprA1 unfolds and forms a helix with SprA1_AS_. Surprisingly, gel retardation assays and mutational analysis revealed that by imperfect base-pairing, the non-overlapping region of SprA1_AS_ binds the antitoxin to the RBS, preventing ribosome loading and translation. Therefore, in a *trans*-encoded manner, the *cis*-encoded asRNA SprA1_AS_ negatively regulates the translation of its cognate antitoxin. A similar *cis-trans* mechanism of action was also reported in the SprA2/SprA2_AS_ type I TA module (Germain-Amiot et al., 2019).

The SprG/F TA module is another intriguing example of the TA system, in which, the RNA antitoxin can acts in *trans* and exert several functions. This system was originally characterized by its perfect base complementarity between the 3' end of each RNA and for not competing with ribosome loading (Pinel-Marie et al., 2014). However, the authors showed that this was sufficient to destabilize mRNA and toxic peptide levels, even if the mechanism was not precisely defined. In a more recent study, SprF1 was demonstrated to have a novel *trans*-effect on ribosomes and polysomes (Pinel-Marie et al., 2021). A purine-rich sequence in the antitoxin is responsible for binding ribosomes, which results in global translation initiation interference and increased tolerance to antibiotics, thereby enabling the formation of persister cells. This new finding suggests that type I antitoxin RNA functions are not restricted to regulation of their cognate toxins.

### *Trans*-Encoded sRNAs

*Trans*-encoded sRNAs are associated with intergenic regions and are relatively easy to identify experimentally or *via* predictive tools, since they have distinctive features including a consensus promoter region and U-tract following inverted repeated sequences (intrinsic terminator). Their biogenesis occurs at a locus distant from their targets, so their seed sequences share partial complementarity with them. These sRNAs thus usually regulate more than one mRNA through some of the mechanisms detailed for asRNAs. This partial complementarity, which could represent an apparent weakness, is in the most cases circumvented with the help of RNA chaperones. These sRNAs transcribed from intergenic regions represent the largest set of functionally characterized molecules in the bacterial kingdom. They are expressed under specific conditions to fine-tune gene expression and are highly structured, which may contribute to their increased stability (Waters and Storz, 2009). Typically, they have one or more stem-loops, some of which have cytosine-rich motifs that favor interaction with the RBSs of their targets (Geissmann et al., 2009). As mentioned above, *trans*-encoded sRNAs are involved in a large set of functions, with repression the most common outcome, although examples of activation have also been reported.

#### *Trans*-Encoded sRNA Activators

**Figure 5:**
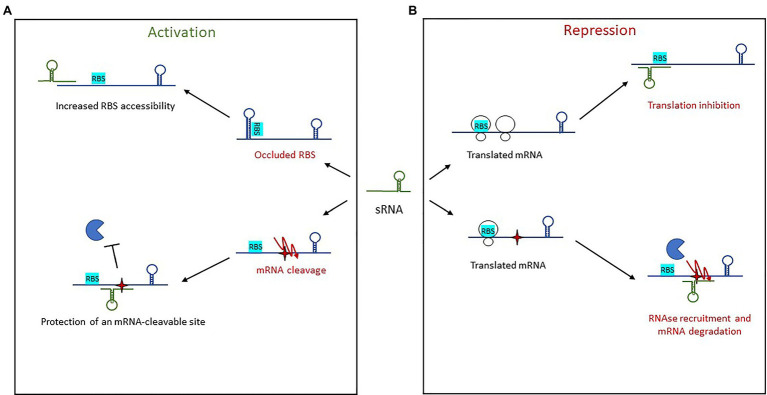
Mechanisms of action used by *trans*-encoded sRNAs. **(A)** In post-transcriptional activation, sRNA binding of an mRNA target results in either increased RBS accessibility or protection of an mRNA-cleavable site. **(B)** Repression of gene expression by *trans*-encoded sRNAs occurs when the sRNA binds an mRNA target, resulting either in translation inhibition by preventing ribosome loading or else recruitment of an RNase for mRNA degradation.

While considered marginal, there are growing examples of *trans*-encoded sRNAs that permit increased translation and/or mRNA stabilization (Figure 5A). Activation of sRNA base-pairing is typically associated with the 5' UTR, although targeting of the coding sequence is also possible (Papenfort and Vanderpool, 2015).

Activation *via* the 5' UTR involves two mechanisms of action. The first is an anti-antisense mechanism that induces structural modifications to enable the unfolding of an intrinsic structure that inhibits translation. This leads to the release of the occluded RBS, thus enhanced ribosomal access. This has been observed with RNAIII in *S. aureus* (Morfeldt et al., 1995), further discussed below, as well as with Rli27 in *L. monocytogenes* (Quereda et al., 2014). Rli27 is involved in cell-wall formation during a pathogen’s intracellular lifestyle. It activates the cell wall-encoding protein Lmo0514 *via* binding of its 5' UTR, thus unmasking the RBS and promoting Lmo0514 production inside eukaryotic cells. The second mechanism of 5' UTR-activation is mRNA stabilization, an even a less-described phenomenon in which sRNA target-binding protects it from RNases and therefore from cleavage. This is seen with RydC in *Salmonella* (Fröhlich et al., 2013). There, RydC adopts a pseudoknot structure at its 5' end that contains a seed sequence involved in recognizing a 5' UTR located far upstream from the RBS of *cfa* mRNA, which encodes CFA synthase. This seed pairing activates Cfa expression by mRNA stabilization through the mRNA cleavage protection provided by RNase E. In *Clostridium perfringens*, another mechanism of action was reported for the stabilization of the mRNA *colA*, which encodes collagenase toxin (Obana et al., 2010). In this pathogen, the 3' region of VR-RNA binds *colA* to mediate cleavage of the mRNA 78 nt upstream the A of initiation codon, resulting in the release of a more stable mRNA.

Other sRNAs activate translation by base-pairing with the coding sequence. In *E. coli* and *Salmonella*, the sRNA SgrS involved in sugar homeostasis displays several distinctive features (Papenfort et al., 2013). Like RNAIII, it has dual functions, acting both as a *trans*-encoded sRNA as well as encoding the small protein SgrT. Among the known targets of SgrS is the second gene of the bicistronic operon *pldB-yigL*, which is under its positive control and encodes a phosphatase. Under normal conditions, the transcript is processed by RNase E, resulting in *yigL* transcript level adjustments. When the concentration of phosphorylated sugars increases, regulation is needed to avoid cell toxicity, so SgrS is expressed. It binds the coding sequence to stabilize the *yigL* transcript by masking a cleavage site recognized by RNase E, thereby preventing its degradation. The increased phosphatase activity lowers sugar phosphate levels and causes the excretion of non-phosphorylated sugars. At the same time, SgrS effectively controls sugar phosphate levels by repressing the expression of three other targets involved in sugar transport, once again demonstrating the versatility of sRNAs as regulators.

#### *Trans*-Encoded sRNA Repressors

Repression by *trans*-encoded sRNAs involves transcription attenuation, translation repression, and mRNA degradation (Figure 5B). Regulatory signals are often contained within the 5' UTR of target mRNAs. The most common mechanism of action in this group is the repression of translation initiation, with sRNAs binding the RBS to prevent loading by the 30S ribosomal subunit. This binding sometimes encompasses the AUG codon, although any interaction surrounding the RBS and AUG up to five codons into the coding sequence is inhibitory (Hüttenhofer and Noller, 1994; Bouvier et al., 2008). Several seed regions/sequence patterns have been described for these *trans*-encoded sRNAs, and the association of single-stranded RNAs to form heteroduplexes is not the only one. The patterns usually reflect the structural shape of RNAs and include several hairpin loops that require additional features. For instance, “kissing complexes” (loop–loop interactions between sRNAs and mRNAs) can occur when GC-rich sequences present in the sRNA loop associate with the RBS of their target (Lioliou et al., 2010).

To facilitate complex formation, sRNAs (and sometimes mRNAs) have to unfold to generate double-stranded heteroduplexes, which are thermodynamically favorable. C-rich conserved sequences were identified in stem-loops and shown to be involved in the recognition of G-rich sequences such as the RBSs of mRNA targets. This was first proved in *E. coli* for the repression of *fhlA* by OxyS (Altuvia et al., 1998), then later on in multiple pathogens including *L. monocytogenes*, *Salmonella*, *H. pylori*, *V. cholerae*, and *S. aureus* (Papenfort et al., 2008, 2015; Geissmann et al., 2009; Pernitzsch et al., 2014; Sievers et al., 2014). In *S. aureus*, bioinformatic and phylogenetic analyses were used to identify a conserved unpaired UCCC motif present in the apical loop of several sRNAs, and this was interpreted to be the potential signature of Hfq-independent sRNAs in Gram-positive bacteria (Geissmann et al., 2009; Jørgensen et al., 2020). The role of this motif in the repression of translation was demonstrated for the sRNA RsaE (Geissmann et al., 2009). In *L. monocytogenes*, LhrC sRNAs contain three redundant CU-rich motifs, with one in a stem-loop, another on a single-strand region, and the last occurring in the terminator (Sievers et al., 2014). These sites are important for the translational repression of LapB at the RBS, and another study showed that LhrC translationally represses OppA *via* RBS binding of two of the three CU-rich motifs (Sievers et al., 2015). However, there are also examples of sRNAs blocking translation by masking the RBS with seed sequences that do not exhibit strong C-rich motifs. In *Salmonella*, IsrM, the sRNA involved in virulence, represses the expression of *hilE* and *sopA* mRNAs using two different seeds, which seem to have random C-distributions although they still sequester the RBS (Gong et al., 2011). The presence of two seeds in IsrM indicates that sRNAs can have several regulatory domains. In *S. aureus*, the multifaceted sRNA RsaI expressed under glucose-limited conditions displays similar properties, with two distinct regulatory domains (Bronesky et al., 2019). The first is a typical unpaired CU-rich sequence that base pairs with the RBSs of most of the identified mRNA targets (sugar uptake and metabolism). The second is a G-rich sequence that binds the CU-rich tracts of other sRNAs such as RsaD, RsaG, RsaH, and RsaE, indicating the potential existence of other mechanisms of actions for controlling sRNA functions.

Apart from the ribosome binding site, other target regions do exist. For instance, sRNAs can bind their mRNA targets upstream from the RBS, within the coding region or at the 3' UTR. The *Salmonella* sRNA GcvB contains the GU-rich sequence that interacts with the CA-rich sequences of its target mRNAs (Sharma et al., 2007). For at least one target, *gltl* mRNA, the sRNA specifically recognizes a sequence far upstream from the RBS (~50 nt) that actually acts as a translational enhancer sequence. Still in *Salmonella*, examples of sRNAs binding coding sequences several nucleotides downstream from the RBS were also reported. In that bacterium, MicC sRNAs repress *ompD* mRNAs *via* binding to codons 23–26, which is sufficient for repression (Pfeiffer et al., 2009). By doing so, MicC increases RNase E-dependent *ompD* mRNA decay rather than repressing translation. Interestingly, the SdsR sRNA also represses *ompD* using a similar mechanism between the 15th and 26th codons (Fröhlich et al., 2012).

The sRNA can also bind the 3' UTR of an mRNA to modulate its expression. For instance, *S. aureus* RsaI binds the 3' UTR of the mRNA *icaR*, which encodes a transcriptional repressor of exopolysaccharide production (Bronesky et al., 2019). Although the mechanism of action is yet not understood, this binding contributes to the translational repression of IcaR either by preventing the action of *trans*-acting activators (proteins or RNAs) or by indirectly stabilizing the interaction between the 5' and 3' UTRs of *icaR* mRNAs, known to sequester the RBS.

Along with translation inhibition, sRNA-mediated regulation often involves degradation by RNases. Untranslated mRNAs are subjected to RNA degradation since the absence of polysomes does not protect RNAs from RNAses (Deana and Belasco, 2005). Furthermore, the sRNA-mRNA duplex can be used to recruit RNaseE as the means to control RNA decay (Morita et al., 2005; Bandyra et al., 2012). Some sRNAs are then co-degraded with their targets, some are recycled like enzymes, and the fate of the others depends on their molecular target (Massé et al., 2003; Feng et al., 2015). In addition to RNase E, roles for RNase III and RNase Z were also reported in target RNA degradation (Dutta and Srivastava, 2018).

Altogether, study of *trans*-encoded sRNAs reveals that they can act anywhere on their mRNA targets and that any parts of their sequences can have regulatory functions, implying unlimited possibilities for their mechanisms of action.

### *Cis*-Acting Regulatory Elements of mRNAs: Riboswitches, Thermosensors, and T Boxes

Although not considered to be sRNAs, riboswitches are pivotal players in RNA-mediated regulation of gene expression, and as we will see later, their extraordinary versatility lets them closely link with sRNAs. Riboswitches are natural aptamers usually identified in the 5' UTR of some mRNAs in both Gram-positive and Gram-negative bacteria. They are *cis*-acting elements regulating at the transcriptional and/or translational levels the expression of their downstream genes. To do this, they sense by physical interaction metabolite variations, then modify their secondary structures to form two mutually exclusive RNA conformations (Mironov et al., 2002; Winkler et al., 2002; McDaniel et al., 2003; Nudler, 2006). They contain two distinct functional domains: a metabolite-sensing domain, and an expression platform. Ligand binding induces conformational changes that lead to transcription termination or inhibition of translation initiation. One interesting feature of riboswitches is their conserved sensing domain and the variability of their expression platform. This is the case for the riboswitch that senses thiamine pyrophosphate (TPP): its sensing domain specifically and selectively binds TPP, while its expression platform induces transcription termination in Gram-positive bacteria and suppresses translation initiation in Gram-negative bacteria (Serganov et al., 2004; Nudler, 2006). Another specific feature of riboswitches is their ability to bind ligands without the need to establish Watson-Crick base pairs. The riboswitch folds into a very specific configuration that allows target metabolite recognition and sequestration due to the hydrogen bonds of RNA bases and ribose sugars. Riboswitches are widespread and diverse, as a plethora of effectors have been identified (TPP, SAM, FMN, sugars, divalent ions, etc.). Usually, they control the expression of genes that have functional links with their effector.

The canonical description of the riboswitch mode of action was overturned by the discovery that they can act as RNA thermometers and pH meters, thus they can be active without necessarily requiring metabolite sensing. RNA thermometers generally fold in a temperature-dependent manner to generate alternative conformations that affect translation (Kortmann and Narberhaus, 2012). The first example of an RNA thermometer was identified in the pathogen *L. monocytognes* (Johansson et al., 2002). In this study, the authors showed that the switch into virulence orchestrated by PrfA, the master regulator of virulence, is actually dependent on a conformational change of the 5' UTR of its cognate mRNA. At 30°C, the *prfA* 5' UTR adopts a secondary structure that masks the RBS, acting as a translation repressor. At 37°C, the energy provided by the increased temperature is sufficient to cause a structural switch that enables translation of the TF. Additional thermometers are found in many pathogens such as *Salmonella* Typhi and *S. aureus* (Hussein et al., 2019; Brewer et al., 2021; Catalan-Moreno et al., 2021), and have been extensively reviewed (Loh et al., 2018).

The first 5' UTR element to be identified in the late 1990s was the T-box, which controls the expression of genes involved in amino acid biosynthesis or use. They are typically upstream aminoacyl-tRNA synthetase transcripts, are widely distributed in Gram-positive bacteria, and they respond to the accumulation of uncharged tRNAs when cognate amino acid concentrations are too low. Contrary to metabolite-sensing riboswitches, T-boxes bind uncharged tRNAs by base-pairing with their anticodons and with the acceptor-end T/D loops to stabilize an anti-terminator element, allowing synthesis of full-length mRNA. In *Clostridium acetobutylicum* (André et al., 2008) and later in *L. monocytogenes* (Mellin et al., 2013), exploration of T-box riboswitches led to the discovery of a novel mechanism of action, with *cis* elements involved in antisense RNA control located downstream (these turned out to be SAM riboswitches) and on the opposite strand of the gene. Other unexpected riboswitch mechanisms of action is reviewed here (Mellin and Cossart, 2015).

## Unconventional Regulatory RNA Biogenesis and Functions

The term “sRNA” was often restricted to those species transcribed from intergenic regions, or as antisense to a coding sequence. Ongoing efforts to identify novel sRNAs and to decipher their mechanisms of action has demonstrated that they harbor an even broader than expected variety of structural traits, biological functions (and even sometimes dual functions), and modes of biogenesis. For instance, studies have shown that RNA regulators are also produced from 5' to 3' UTRs (Miyakoshi et al., 2015b; Heidrich et al., 2017; Carrier et al., 2018).

### The Fascinating Case of RNAIII in *Staphylococcus aureus*

**Figure 6:**
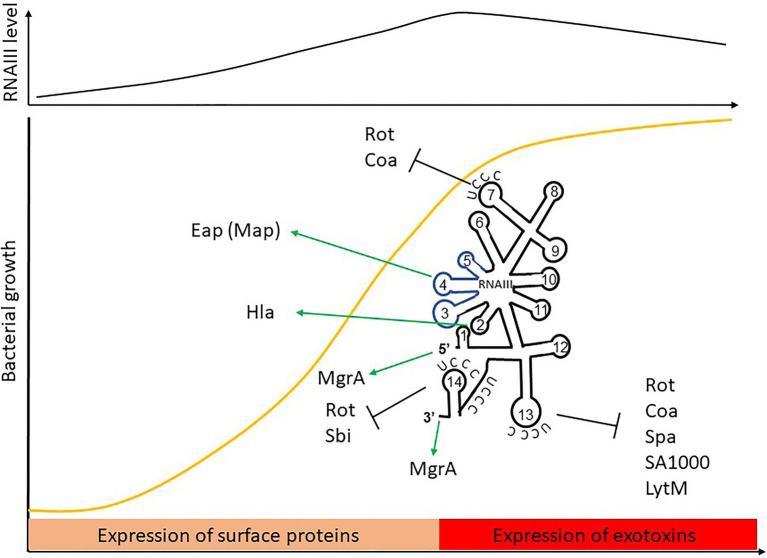
The RNAIII paradigm: activation and repression of gene expression is carried out by a dual-function sRNA encoding δ-hemolysin. The *hld* coding sequence is blue.

RNAIII is one of the best-characterized sRNAs in pathogens and probably even in bacteria. It was first described in 1993, when it was defined as the intracellular effector of the Agr system (Novick et al., 1993). It is one of the most representative examples of the sRNA with distinctive futures. RNAIII is a 514-nt long dual-function sRNA whose expression is regulated by growth phase and cell density through QS. RNAIII acts at a post-transcriptional level by using antisense mechanisms, which lead to translation inhibition and/or modification of target RNA stability (Figure 6). RNAIII positively or negatively regulates a large number of targets through various mechanisms, and has a complex structure with 14 stem-loops (Benito et al., 2000) of which three contain C-rich sequences. These three hairpins, H7, H13, and H14, are involved in translation repression, preventing ribosomes from loading onto target RBSs. The first stem-loop (H1) is required for RNAIII stability, while H14 acts as an intrinsic transcription terminator. H3, H4, and H5 are responsible for the definition of this sRNA as dual-function, another sRNA subgroup which has been extensively reviewed (Gimpel and Brantl, 2017; Raina et al., 2018). These three stem-loops encode δ-hemolysin (Hld), which is a toxin made of 26 amino acids arranged into an α-helix structure, and which permeabilizes host cells (Figure 6). Interestingly, there is not a perfect transcription-translation coupling between *hld* mRNA biogenesis and translation of the Hld peptide. Although the cause for this is not completely understood, it was reported that deletion of the 3' end of RNAIII abolishes this delay, suggesting either that a third party is involved (Balaban and Novick, 1995) or that the 5' and 3' regions of RNAIII are (or serve as) *cis*-regulatory elements. RNAIII is under the positive transcriptional control of AgrA and accumulates in bacteria over the course of the growth phase, reaching its maximal transcript levels during the post-exponential phase (Dunman et al., 2001). With its versatile effects, RNAIII orchestrates the transition between colonization and infection by repressing early virulence genes and activating other virulence factors (Table 1).

#### RNAIII Target Repression

RNAIII represses the expression of several genes involved in virulence (adhesion or immune evasion), including Rot TF (Table 1). The mechanism of action is always translation repression, with RBS binding in order to prevent ribosome loading and translation initiation. In most cases, duplex formation is followed by rapid degradation of the mRNA target through the recruitment of RNase III (Bronesky et al., 2016), although the mechanism differs for the regulation of Sbi and perhaps also for LytM (Boisset et al., 2007; Chabelskaya et al., 2014).

Repression of Spa involves the 3' end domain of RNAIII (Huntzinger et al., 2005). Spa is one of the major surface proteins involved in host interactions as well as virulence (Foster, 2005). Based on sequence complementary, the deletion of RNAIII’s H13 stem-loop abolishes regulation by RNAIII. This repressive hairpin sequesters the *spa* RBS to prevent translation initiation, although efficient repression requires RNase III. A similar repression mechanism involving H13 and RNase III was reported for SA1000, which encodes a fibrinogen-binding protein (Boisset et al., 2007). Repression of Rot, the repressor of toxin Rot requires hairpins H7, H13, and H14, who form kissing complexes to permit translation repression and RNase III cleavage (Boisset et al., 2007). To do this, H7 and H13 enable the formation of a loop–loop interaction with the 5' UTR of *rot*, while H14 is involved in a loop–loop interaction with the mRNA RBS. Two loop–loop interactions are needed for efficient translation inhibition, and binding of RNAIII with *rot* induces a specific signature for RNase III cleavage. Rot is known to inhibit the production of several exotoxins after their transcription as well as to enhance Spa expression. Therefore, RNAIII inhibition of Rot indirectly promotes exotoxin expression and represses surface protein production.

As with Rot, RNAIII repression of Coa involves two stem-loops (H7 and H13), although the structural conformations differ between these examples (Chevalier et al., 2010). In this case, H13 sequesters the *coa* RBS through canonical imperfect double-strand base-pairing and this is sufficient to block the formation of ribosomal initiation complexes. Additionally, H7 forms a loop–loop interaction with the 3' end of *coa*, and both RNAIII-*coa* complexes serve as templates for RNase III.

RNAIII regulation of LytM is less documented. While bioinformatic predictions suggest that stem-loop H13 is involved (Boisset et al., 2007), and it is known that the *lytM* RBS is an RNAIII target (Chunhua et al., 2012), the precise mechanism of action and the putative involvement of RNase have not yet been determined.

Sbi is another target repressed by RNAIII (Chabelskaya et al., 2014), and it is negatively regulated by SprD sRNAs (Chabelskaya et al., 2010). Sbi protein participates in anti-opsonization effects by binding immunoglobulins (Foster, 2005). Three distant RNAIII domains interact with *sbi* mRNAs, with two annealing regions located within the *sbi* coding region and one on the RBS to block translation initiation. Since RNAIII and SprD are not expressed at the same time, these studies highlight a cooperative role of these two sRNAs to precisely control Sbi expression suggesting the presence of complex sRNA control networks in bacteria.

#### RNAII Activation of Gene Expression

In addition to its inhibitory roles, RNAIII can also directly activate several targets. This is true in the case of Hla, whose expression is reduced in a mutant lacking RNAIII (Morfeldt et al., 1995). RNAIII binds *hla* mRNAs to prevent the formation of a structure that would sequester the RBS, thereby promoting RBS accessibility and translation. Unlike the mechanism involving repression, here the 5' end (H2 in RNAIII) is required for base-pairing with *hla*. Map (also known as Eap) is another target that is stimulated by RNAIII (Liu et al., 2011). To activate Map expression, RNAIII binds the 5' UTR of *map* using its hairpin H4, which suggests a structural modification that enables ribosomal loading, although the mechanism of action has not yet been unraveled. RNAIII was also shown to positively regulate MgrA (Gupta et al., 2015), the TF which inhibits surface proteins, autolysis, and biofilm formation, as well as promoting capsule synthesis (Bronesky et al., 2016). Interestingly, MgrA also activates the expression of Agr and thus indirectly RNAIII (Ingavale et al., 2005). RNAIII stabilizes *mgrA* RNAs by double-strand base-pairing using both its 5' and 3' ends (Bronesky et al., 2016), with each end interacting simultaneously with the 5' UTR of *mgrA* mRNA to allow mRNA stabilization and translation.

RNAIII thus differs from the conventional dogma about sRNAs in many ways. It contains a coding sequence, and is therefore considered as a dual-function sRNA. It is longer than most sRNAs, and has a complex structure containing several hairpins, which are crucial for gene repression. Its gene repression mechanism involves translation initiation control and is often associated with RNase III cleavage. The 5' end is thought to be involved in gene activation *via* mRNA stabilization and translation activation, while the 3' end is involved in both activation and the repression mechanisms. The 3' end is also important for RNAIII’s own stability, and therefore may be a key *cis* element for Hld production. Finally, little is known regarding the role of the other hairpins, suggesting that there may be additional features left to discover.

### 5' UTR-Derived sRNAs

5' UTRs are normally considered to be riboswitches, *cis*-acting elements. The first major evidence of the sRNA produced from a 5' UTR was the cleavage of the two SAM riboswitches SreA and SreB in *L. monocytogenes* (Loh et al., 2009). In that report, the biogenesis of SreA and that sRNA’s mechanism of action were described extensively. During methionine starvation, SAM concentrations are relatively low, leading to the formation of an anti-terminator structure that enables the transcription of the downstream gene. When the concentrations of this ligand increase along with methionine biosynthesis, SAM binds the riboswitch. This allows structural rearrangement and transcription termination, and results in the accumulation of SreA, an sRNA transcript of 229 nts which has *trans*-acting features. Remarkably, SreA controls the expression of the virulence TF PrfA by binding the 5' UTR of its mRNA, while PrfA exerts a positive control on SreA expression. This shows that some *cis*-acting regulators can be cleaved to act in *trans* in response to environmental cues. Another example of a riboswitch-producing *trans*-acting sRNA was reported in *L. monocytogenes* (Mellin et al., 2014). The authors showed that in the absence of the ligand, a riboswitch binding vitamin B12 is cleaved and releases Rli55. In turn, Rli55 expression prevents the expression of the *eut* genes involved in ethanolamine use and whose expression depends on vitamin B12 availability. To do this, Rli55 sequesters EutV, a regulatory protein which binds *eut* mRNAs to prevent premature termination of transcription. A similar mechanism for regulating *eut via* protein sequestration was described in *E. faecalis*, although there an adenosyl cobalamine-binding riboswitch releases EutX *trans*-acting sRNAs (DebRoy et al., 2014). In *S. aureus*, the sRNA Teg49 is derived from the 5' UTR region of TF *sarA* mRNA (Beaume et al., 2010; Kim et al., 2014). The expression of *sarA* is under the control of the three promoters P1 through P3, with P3 important for expression during the post-exponential growth phase. Teg49 is transcribed from P3 and is probably the result of RNase III processing. Another study indicated a role of Teg49 in virulence, although the mechanism of action is not clearly understood (Manna et al., 2018).

Based on the first discoveries, 5' UTR-derived sRNAs are probably more present in Gram-positive pathogens. It was proposed that the relative absence of such sRNAs in Gram-negative pathogens is explained by the fact that most sRNAs studies have been designed based on Hfd-binding. This binding usually occurs with the 3' poly(U) tail of sRNAs located downstream from intrinsic terminators, and these are likely to be absent in 5' UTR-derived sRNAs. Additionally, the need for Hfq-binding was questioned (Carrier et al., 2018). The mystery was solved in *P. aeruginosa* with a screen that attempted to map transcription termination sites affected by homoserine lactone quorum sensing (Thomason et al., 2019). Term-seq analysis identified RhlS as a novel Hfq-dependent sRNA expressed from the 5' UTR of *rhll*, an actor in the Rhl two-component system related to quorum sensing. Interestingly, RhlS is not derived from a riboswitch, but is induced when homoserine lactone concentrations increase. RhlS not only controls the translation of its downstream gene (*rhll*), but also regulates the translation of *fpvA* mRNAs transcribed from a distant locus and which encode a siderophore pyoverdine receptor, although the mechanism of action here is still unclear. Finally, recent work on *E. coli* focusing on the identification of 3' ends from 5' UTRs revealed the presence of *trans*-acting sRNA sponges (Adams et al., 2021).

### 3' UTR Is a Reservoir of *Trans*-Acting sRNAs

Expression of 3' UTR-derived sRNAs occurs frequently in bacteria, and the number of examples is growing quickly. Over the past few years, their identification has become easier with the development of high-throughput screenings such as Term-seq, RIL-seq, RIP-seq, and CLASH (Dar et al., 2016; Melamed et al., 2016; Hoyos et al., 2020). These sRNAs act in various physiological regulons including amino acid transport and biogenesis. They are categorized into two classes depending on whether they have their own promoters (type I) or if they are produced by mRNA processing (type II; Miyakoshi et al., 2015b). While type I sRNAs seem completely independent of the gene encoded upstream when it comes to biogenesis the functions they regulate, the expression of type II sRNAs depends on the initial mRNA transcription. So far, more type II molecules have been discovered, and most 3' UTR-derived sRNAs have been discovered in Gram-negative bacteria. Post-transcriptional regulation by 3' UTR elements was recently reviewed (Menendez-Gil and Toledo-Arana, 2020).

#### Type I 3' UTR-Derived sRNAs

This type of 3' UTR-derived sRNA contains its own promoter, located either within the coding sequence or immediately downstream. In 2012, Hfq co-immunoprecipitation of transcripts followed by RNA-seq analysis enabled the discovery of novel sRNAs whose RNA sequences mapped with the 3' region of mRNAs (Chao et al., 2012). During this screen, the authors identified DapZ, an 80-nt sRNA transcribed from its own promoter and that overlaps the *dapB* 3' UTR. Although they share the same terminator, their transcription is independent of each other. DapZ modulates the synthesis of ABC transporters of Opp and Dpp by base-pairing with their cognate mRNAs. One interesting feature of DapZ is that it contains a repressing seed sequence (G/U-rich domain) similar to that of GcvB sRNA, a global regulator of amino acid transport and biosynthesis genes, so they recognize the same targets. This indicates that despite being independent, the two sRNAs can have complementary functions.

In *S. aureus*, a search for novel transcripts uncovered an intricate organization in the Newman strain, with an unusual condensed sRNA cluster (Srn_9342 to Srn_9346; Bronsard et al., 2017). Using 5' RACE mapping combined with TEX and polyphosphatase treatments, the authors were able to show that the Srn_9342 transcription start site is located within the coding region of the upstream gene. This was supported by the identification of a putative sigma B binding site (Mader et al., 2016). Interestingly, Srn_9342 is expressed in two forms having different lengths and sharing the same 5' extremity, although the mechanism of biogenesis of each form and their respective functions remain to be elucidated. In this specific example, two terminators were predicted (Bronsard et al., 2017), with one sharing the upstream coding sequence as occurs in typical type I 3' UTR-derived sRNAs, and the other specific to the long sRNA species, indicating another variation in sRNA biogenesis within this category.

#### Type II 3' UTR-Derived sRNAs

This type of sRNA is derived from RNase E cleavage of an mRNA. An example is CpxQ, a ~60 nt-long transcript generated through cleavage of the 3' UTR of *cpxP* mRNA and which requires Hfq for optimal maturation as well as protection from further RNA decay (Chao and Vogel, 2016). Both CpxQ sRNAs and CpxP proteins are involved in inner membrane homeostasis, with the protein acting as a chaperone for misfolded proteins in the periplasm, and the sRNA controlling their expression in the cytosol. Thus, unlike with type I 3' UTR-derived sRNAs, CpxQ synthesis depends on *cpxP* transcription, and these two elements cooperate to carry out the same function. In *S. aureus*, after cleavage by the double-stranded RNase III, the sRNA RsaC is released from the 3' UTR of the *mntABC* operon, which encodes the major manganese ABC transporter (Lalaouna et al., 2019). This sRNA is intriguing, as its length varies between isolates due to the presence of a variable number of repeats within its internal RNA sequence. The 3' domain of RsaC was found to be involved in translational repression of *sodA* mRNA during manganese starvation, thereby modulating the response to oxidative stress.

The regulatory functions of these 3' UTR-derived sRNAs are often linked to the biological roles of their associated upstream genes (Menendez-Gil and Toledo-Arana, 2020). They therefore autoregulate the expression of their associated genes at the post-transcriptional level, inhibit translation by base-pairing, and serve as negative feedback controls (Chao and Vogel, 2016; Miyakoshi et al., 2019; Hoyos et al., 2020; Wang et al., 2020). As the description of this sub-class of sRNAs is relatively new, it is expected that its ranks will increase in the future.

### sRNAs Excised/Expressed From Coding Sequences

While the examples cited above pertain to biogenesis from the 3' UTR or at least from the 3' end of mRNA coding regions, in-depth study of the transcriptomes of *E. coli*, *Enterobacter aerogenes*, and *Salmonella* Typhimurium uncovered the biogenesis of decay-generated noncoding RNAs (decRNAs) from coding regions situated far from mRNA 3' ends (Dar and Sorek, 2018). The authors identified a set of conserved sequences generated through RNase E activity and predicted to interact with the RNA chaperones Hfq and ProQ. A search for transcriptional start sites using 5' RNA-seq mapping uncovered numerous sRNAs transcribed from within coding sequences in several species including *Borrelia burgdorferi* and *Leptospira interrogans* (Adams et al., 2017; Zhukova et al., 2017). However, the functions of this novel type of sRNAs remain to be elucidated.

### sRNA Processing to Produce Novel sRNAs or Additional Functions

In *S. aureus*, Srn_9342 is not the only sRNA transcribed into two forms (Bronsard et al., 2017), and similar properties were observed for both RsaA (Romilly et al., 2014) and RsaE (Schoenfelder et al., 2019). While transcriptional readthrough was just hypothesized for Srn_9342 and RsaA, post-transcriptional modification was actually demonstrated for RsaE. In *S. epidermidis*, RsaE is normally transcribed as a transcript ~110-nt long, but it can undergo processing to release a transcript of ~80 nt which lacks the 5' end. Biogenesis of this second form enables the expansion of the original RsaE targetome. The full-length sRNA specifically regulates antiholin-encoding *lrgA* mRNA by binding the RBS, preventing ribosome loading, and influencing mRNA levels. Conversely, the processed RsaE interacts with the 5' UTR of *icaR* and *sucCD* mRNAs, which encode the IcaR repressor of biofilm formation and the succinyl-CoA synthetase of the TCA cycle, respectively.

### sRNA Sequestration by Other RNAs (Sponge RNAs)

As for any genes, we expect sRNA genes and transcripts to require fine regulation. Several TFs were identified as regulators of sRNA transcription, but regulation or sequestration of sRNAs by other RNAs is a relatively new topic of investigation. Research into type II 3' UTR-derived sRNAs engendered the concept of bacterial sponge RNAs, which soak up other sRNAs rather than just proteins in order to suppress their regulatory functions (Azam and Vanderpool, 2015). The first sponge to be characterized was chb, which sequesters the sRNA ChiX (Figueroa-Bossi et al., 2009). ChiX represses the expression of ChiP, a porin involved in the uptake of chitosugars. In the absence of chitosugars, ChiX represses ChiP since it is not needed. When chitosugars are present, the *chBCARFG* operon is transcribed, and an intercistronic spacer sequence between *chbB* and *chbC* acts as a sponge for ChiX, allowing ChiP expression. Later on, a study reported the case of SroC, a type II 3' UTR-derived sRNA that activates up to 26 targets, while repressing 14, an unusually high number of targets (Miyakoshi et al., 2015a). The authors showed that SroC acts as a sponge of GcvB, the sRNA involved in amino-acid metabolism, and the most repressed of the SroC targets. The intriguing feature is that SroC is produced from the *gltJKL* operon, an mRNA target of GcvB, indicating an elegant cross-talk mechanism. When produced, SroC binds GcvB by base-pairing, allowing the recruitment of RNase E and subsequent GcvB degradation and derepression of its targetome. In other words, GcvB sequestration permits the indirect activation of most of the 26 targets identified in the screen, indicating that sRNAs can function using mechanisms similar to some TFs that repress other repressors to activate gene expression. In a second study, SroC was shown to sequester another sRNA, MgrR, by binding its seed sequence (Acuña et al., 2016). In addition to this sponge activity, SroC also indirectly regulates the expression of other targets functionally related to the GcvB targetome (Miyakoshi et al., 2015a). Interestingly, GcvB is also controlled by the AgvB bacteriophage-encoded sponge RNA (Tree et al., 2014), which adds another layer of complexity: a sponge RNA can mitigate several sRNAs, and a single sRNA can be sequestered by more than one sponge RNA.

Besides 3' UTR mRNA-related biogenesis, sRNA sponges can also be generated from diverse loci such as tRNA transcripts, either mature or precursor tRNA (Lalaouna et al., 2015). During the maturation of the *glyW-cystT-leuZ* polycistronic pre-tRNA, a 3' external transcribed spacer sequence (3' ETS^leuZ^) is released and acts as a sponge for RyhB and RybB sRNAs to reduce transcriptional noise during non-inducing conditions. RybB is also sponged by RbsZ, the sRNA associated with Hfq and ProQ (Melamed et al., 2020). Recent studies in the model organism *E. coli* demonstrated the presence of sRNA sponges ChiZ and IspZ deriving from 5' UTRs (Adams et al., 2021), suggesting that sponge RNAs can be produced from any part of the genome. They can thus be produced independently since they contain their own promoters, they can be processed from an existing transcript, they may belong to intergenic spacer regions or intergenic tRNA spacers, or they can be generated from 3' UTRs (Denham, 2020).

With the concept of sponge, it appears that the bioavailability of the mRNA target and the presence of a sponge RNA are important parts of sRNA-mediated gene regulation. The discovery of sRNA sponges is still recent, and more breakthroughs are expected in the near future, which should help us understand their importance in regulatory networks.

### Mediation of Transcription Termination

In *E. coli* (and perhaps in all bacteria), sRNAs can affect gene expression through a variety of mechanisms of action. Another example of this extraordinary diversity is the ability of some sRNAs to mediate transcription termination. Study of the transcriptional regulation of the *chiPQ* operon by ChiX sRNAs revealed that by pairing with the 5' end of its mRNA target, the sRNA induces Rho-dependent transcription termination (Bossi et al., 2012). The actual mechanism of action relies on the inhibition of ribosome binding, decreasing ribosomes at the Rho utilization site thus increasing Rho-dependent transcription termination. Conversely, other examples of positive regulation have been reported, with DsrA, ArcZ, and RprA regulating *rpoS* expression by preventing Rho from binding to the mRNA, while the sRNAs continue to bind the 5' UTRs (Sedlyarova et al., 2016). Transcription termination regulation by sRNAs was recently reviewed (Chen et al., 2019; Bossi et al., 2020).

### Clusters of Regularly Interspaced Short Palindromic Repeat

Although, there has been an explosion of information about sRNA modes of action and expression, the general rule was that since RNA is the product of DNA transcription, it only rarely retroactively affects DNA. This dogma has been overturned with the discovery of clusters of regularly interspaced short palindromic repeats (CRISPRs), which provide immunity to bacteria by recognizing any re-invasion of nucleic acids (Barrangou et al., 2007). In CRISPR, a guide RNA (crRNA) is crucial for the recognition of foreign DNA or RNA (from bacteriophages, plasmids, or mobile genetic elements), enabling its cleavage by the Cas9 nuclease (Brouns et al., 2008). Following processing, the foreign sequence is integrated into the genome in a CRISPR array usually located close to the Cas system. The CRISPR array is composed of spacer sequences corresponding to foreign DNAs serving as immune memory, and which are flanked by DNA repeats. The DNA-encoded CRISPR system thus involves RNA-mediated recognition of foreign nucleic acids, and functions as a defense system.

## Concluding Remarks

Over the past few decades, a tremendous shift has taken place in how we define RNA species. Although, they were long dismissed as simple messengers, the first in-depth characterizations of non-coding RNAs have paved the way for fascinating discoveries about their functions, which range from essential roles in translation machinery to global regulatory functions. Genome-wide and high-throughput screenings have enabled a rapid evolution in knowledge, and the establishment of new rules. They helped reveal an extraordinary diversity in types of non-coding RNAs and showed that they use an abundance of mechanisms of action. Non-coding RNAs include rRNAs, tRNAs, 6S RNA, ribozymes, riboswitches, CRISPRs, and sRNAs. In terms of viability, sRNAs are often non-essential, but they are valuable for rapid and cost-effective regulation of gene expression in response to environmental cues. Therefore, they appear to be key players, adding another layer to gene expression control. Within sRNAs, a large number of classes and sub-classes have been created as a result of novel discoveries about their genomic locations, biogenesis, and modes of action, and these go way beyond protein classifications based solely on function and domain presence. In each sRNA category, there are a variety of mechanisms of action (with some appearing in several categories), and a large diversity of functions. The boundaries between sub-classes, between sRNAs and riboswitches or CRISPRs, sometimes appear very thin, particularly as novel mechanisms of action or biogenesis are constantly discovered. It is now accepted that their biogenesis can occur from any locus in the genome or in plasmids, and from any type of RNA molecule. Although categorization into multiple and highly specific types permits the organization of information, it may be detrimental for a rapid and simple presentation of what constitutes the sRNA. Cech and Steitz (2014) attempted to summarize the definition of sRNAs based on their most general function, base-pairing with mRNAs to regulate gene expression. However, this definition is limited, as it does not include protein-binding sRNAs. Perhaps just minor adjustments are enough to ensure consensus around a definition expansive enough to avoid obsolescence. Bacterial sRNAs are any RNA molecules that interact with other actors to regulate gene expression.

## Author Contributions

BF and YA planned the outline of the manuscript. YA wrote the review. All authors contributed to the article and approved the submitted version.

## Conflict of Interest

The authors declare that the research was conducted in the absence of any commercial or financial relationships that could be construed as a potential conflict of interest.

## Publisher’s Note

All claims expressed in this article are solely those of the authors and do not necessarily represent those of their affiliated organizations, or those of the publisher, the editors and the reviewers. Any product that may be evaluated in this article, or claim that may be made by its manufacturer, is not guaranteed or endorsed by the publisher.

## References

[ref1] Acuña L. G. Barros M. J. Peñaloza D. Rodas P. I. Paredes-Sabja D. Fuentes J. A.. (2016). A feed-forward loop between SroC and MgrR small RNAs modulates the expression of eptB and the susceptibility to polymyxin B in *Salmonella* Typhimurium. Microbiology 162, 1996–2004. 10.1099/mic.0.000365, PMID: 27571709

[ref2] Adams P. P. Baniulyte G. Esnault C. Chegireddy K. Singh N. Monge M.. (2021). Regulatory roles of *Escherichia coli* 5' UTR and ORF-internal RNAs detected by 3' end mapping. elife 10:e62438. 10.7554/eLife.62438, PMID: 33460557 PMC7815308

[ref3] Adams P. P. Flores Avile C. Popitsch N. Bilusic I. Schroeder R. Lybecker M.. (2017). In vivo expression technology and 5' end mapping of the *Borrelia burgdorferi* transcriptome identify novel RNAs expressed during mammalian infection. Nucleic Acids Res. 45, 775–792. 10.1093/nar/gkw1180, PMID: 27913725 PMC5314773

[ref4] Alix E. Blanc-Potard A.-B. (2008). Peptide-assisted degradation of the *Salmonella* MgtC virulence factor. EMBO J. 27, 546–557. 10.1038/sj.emboj.7601983, PMID: 18200043 PMC2241655

[ref5] Altman S. Baer M. F. Bartkiewicz M. Gold H. Guerrier-Takada C. Kirsebom L. A.. (1989). Catalysis by the RNA subunit of RNase P—a mini review. Gene 82, 63–64. 10.1016/0378-1119(89)90030-9, PMID: 2479591

[ref6] Altuvia S. Zhang A. Argaman L. Tiwari A. Storz G. (1998). The *Escherichia coli* OxyS regulatory RNA represses fhlA translation by blocking ribosome binding. EMBO J. 17, 6069–6075. 10.1093/emboj/17.20.6069, PMID: 9774350 PMC1170933

[ref7] André G. Even S. Putzer H. Burguière P. Croux C. Danchin A.. (2008). S-box and T-box riboswitches and antisense RNA control a sulfur metabolic operon of *Clostridium acetobutylicum*. Nucleic Acids Res. 36, 5955–5969. 10.1093/nar/gkn601, PMID: 18812398 PMC2566862

[ref8] Arthur D. C. Ghetu A. F. Gubbins M. J. Edwards R. A. Frost L. S. Glover J. N. M. (2003). FinO is an RNA chaperone that facilitates sense-antisense RNA interactions. EMBO J. 22, 6346–6355. 10.1093/emboj/cdg607, PMID: 14633993 PMC291848

[ref9] Asano K. Mizobuchi K. (1998a). An RNA pseudoknot as the molecular switch for translation of the repZ gene encoding the replication initiator of IncIalpha plasmid ColIb-P9. J. Biol. Chem. 273, 11815–11825. 10.1074/jbc.273.19.11815 9565606

[ref10] Asano K. Mizobuchi K. (1998b). Copy number control of IncIalpha plasmid ColIb-P9 by competition between pseudoknot formation and antisense RNA binding at a specific RNA site. EMBO J. 17, 5201–5213. 10.1093/emboj/17.17.5201 9724656 PMC1170848

[ref11] Attaiech L. Boughammoura A. Brochier-Armanet C. Allatif O. Peillard-Fiorente F. Edwards R. A.. (2016). Silencing of natural transformation by an RNA chaperone and a multitarget small RNA. Proc. Natl. Acad. Sci. U. S. A. 113, 8813–8818. 10.1073/pnas.1601626113, PMID: 27432973 PMC4978251

[ref12] Augagneur Y. King A. N. Germain-Amiot N. Sassi M. Fitzgerald J. W. Sahukhal G. S.. (2020). Analysis of the CodY RNome reveals RsaD as a stress-responsive riboregulator of overflow metabolism in *Staphylococcus aureus*. Mol. Microbiol. 113, 309–325. 10.1111/mmi.14418 31696578

[ref13] Augagneur Y. Wesolowski D. Tae H. S. Altman S. Ben Mamoun C. (2012). Gene selective mRNA cleavage inhibits the development of *Plasmodium falciparum*. Proc. Natl. Acad. Sci. U. S. A. 109, 6235–6240. 10.1073/pnas.1203516109 22474358 PMC3341008

[ref14] Avila-Calderón E. D. Araiza-Villanueva M. G. Cancino-Diaz J. C. López-Villegas E. O. Sriranganathan N. Boyle S. M.. (2015). Roles of bacterial membrane vesicles. Arch. Microbiol. 197, 1–10. 10.1007/s00203-014-1042-7, PMID: 25294190

[ref15] Azam M. S. Vanderpool C. K. (2015). Talk among yourselves: RNA sponges mediate cross talk between functionally related messenger RNAs. EMBO J. 34, 1436–1438. 10.15252/embj.201591492, PMID: 25916829 PMC4474520

[ref16] Babitzke P. Romeo T. (2007). CsrB sRNA family: sequestration of RNA-binding regulatory proteins. Curr. Opin. Microbiol. 10, 156–163. 10.1016/j.mib.2007.03.007, PMID: 17383221

[ref17] Balaban N. Novick R. P. (1995). Translation of RNAIII, the *Staphylococcus aureus* agr regulatory RNA molecule, can be activated by a 3'-end deletion. FEMS Microbiol. Lett. 133, 155–161. 10.1111/j.1574-6968.1995.tb07877.x, PMID: 8566701

[ref18] Balbontín R. Fiorini F. Figueroa-Bossi N. Casadesús J. Bossi L. (2010). Recognition of heptameric seed sequence underlies multi-target regulation by RybB small RNA in *Salmonella enterica*. Mol. Microbiol. 78, 380–394. 10.1111/j.1365-2958.2010.07342.x, PMID: 20979336

[ref19] Bandyra K. J. Said N. Pfeiffer V. Górna M. W. Vogel J. Luisi B. F. (2012). The seed region of a small RNA drives the controlled destruction of the target mRNA by the endoribonuclease RNase E. Mol. Cell 47, 943–953. 10.1016/j.molcel.2012.07.015, PMID: 22902561 PMC3469820

[ref20] Barrangou R. Fremaux C. Deveau H. Richards M. Boyaval P. Moineau S.. (2007). CRISPR provides acquired resistance against viruses in prokaryotes. Science 315, 1709–1712. 10.1126/science.1138140 17379808

[ref21] Bauriedl S. Gerovac M. Heidrich N. Bischler T. Barquist L. Vogel J.. (2020). The minimal meningococcal ProQ protein has an intrinsic capacity for structure-based global RNA recognition. Nat. Commun. 11:2823. 10.1038/s41467-020-16650-6, PMID: 32499480 PMC7272453

[ref22] Beaume M. Hernandez D. Farinelli L. Deluen C. Linder P. Gaspin C.. (2010). Cartography of methicillin-resistant *S. aureus* transcripts: detection, orientation and temporal expression during growth phase and stress conditions. PLoS One 5:e10725. 10.1371/journal.pone.0010725, PMID: 20505759 PMC2873960

[ref23] Benito Y. Kolb F. A. Romby P. Lina G. Etienne J. Vandenesch F. (2000). Probing the structure of RNAIII, the *Staphylococcus aureus* agr regulatory RNA, and identification of the RNA domain involved in repression of protein A expression. RNA 6, 668–679. 10.1017/S1355838200992550, PMID: 10836788 PMC1369947

[ref24] Bessaiah H. Pokharel P. Loucif H. Kulbay M. Sasseville C. Habouria H.. (2021). The RyfA small RNA regulates oxidative and osmotic stress responses and virulence in uropathogenic *Escherichia coli*. PLoS Pathog. 17:e1009617. 10.1371/journal.ppat.1009617 34043736 PMC8205139

[ref25] Bhatt S. Egan M. Jenkins V. Muche S. El-Fenej J. (2016). The tip of the iceberg: on the roles of regulatory small RNAs in the virulence of enterohemorrhagic and enteropathogenic *Escherichia coli*. Front. Cell. Infect. Microbiol. 6:105. 10.3389/fcimb.2016.00105, PMID: 27709103 PMC5030294

[ref26] Bohn C. Rigoulay C. Bouloc P. (2007). No detectable effect of RNA-binding protein Hfq absence in *Staphylococcus aureus*. BMC Microbiol. 7:10. 10.1186/1471-2180-7-10, PMID: 17291347 PMC1800855

[ref27] Bohn C. Rigoulay C. Chabelskaya S. Sharma C. M. Marchais A. Skorski P.. (2010). Experimental discovery of small RNAs in *Staphylococcus aureus* reveals a riboregulator of central metabolism. Nucleic Acids Res. 38, 6620–6636. 10.1093/nar/gkq462, PMID: 20511587 PMC2965222

[ref28] Boisset S. Geissmann T. Huntzinger E. Fechter P. Bendridi N. Possedko M.. (2007). *Staphylococcus aureus* RNAIII coordinately represses the synthesis of virulence factors and the transcription regulator rot by an antisense mechanism. Genes Dev. 21, 1353–1366. 10.1101/gad.423507, PMID: 17545468 PMC1877748

[ref29] Bossi L. Figueroa-Bossi N. Bouloc P. Boudvillain M. (2020). Regulatory interplay between small RNAs and transcription termination factor rho. Biochim. Biophys Acta Gene Regul. Mech. 1863:194546. 10.1016/j.bbagrm.2020.194546, PMID: 32217107

[ref30] Bossi L. Schwartz A. Guillemardet B. Boudvillain M. Figueroa-Bossi N. (2012). A role for rho-dependent polarity in gene regulation by a noncoding small RNA. Genes Dev. 26, 1864–1873. 10.1101/gad.195412.112 22895254 PMC3426764

[ref31] Bouvier M. Sharma C. M. Mika F. Nierhaus K. H. Vogel J. (2008). Small RNA binding to 5’ mRNA coding region inhibits translational initiation. Mol. Cell 32, 827–837. 10.1016/j.molcel.2008.10.027, PMID: 19111662

[ref32] Brantl S. (2002). Antisense-RNA regulation and RNA interference. Biochim. Biophys. Acta 1575, 15–25. 10.1016/s0167-4781(02)00280-4 12020814

[ref33] Brantl S. (2007). Regulatory mechanisms employed by cis-encoded antisense RNAs. Curr. Opin. Microbiol. 10, 102–109. 10.1016/j.mib.2007.03.012, PMID: 17387036

[ref34] Brantl S. Birch-Hirschfeld E. Behnke D. (1993). RepR protein expression on plasmid pIP501 is controlled by an antisense RNA-mediated transcription attenuation mechanism. J. Bacteriol. 175, 4052–4061. 10.1128/jb.175.13.4052-4061.1993, PMID: 8320221 PMC204834

[ref35] Brantl S. Jahn N. (2015). sRNAs in bacterial type I and type III toxin-antitoxin systems. FEMS Microbiol. Rev. 39, 413–427. 10.1093/femsre/fuv003, PMID: 25808661

[ref36] Brewer S. M. Twittenhoff C. Kortmann J. Brubaker S. W. Honeycutt J. Massis L. M.. (2021). A Salmonella Typhi RNA thermosensor regulates virulence factors and innate immune evasion in response to host temperature. PLoS Pathog. 17:e1009345. 10.1371/journal.ppat.1009345, PMID: 33651854 PMC7954313

[ref37] Bronesky D. Desgranges E. Corvaglia A. François P. Caballero C. J. Prado L.. (2019). A multifaceted small RNA modulates gene expression upon glucose limitation in *Staphylococcus aureus*. EMBO J. 38:e99363. 10.15252/embj.201899363, PMID: 30760492 PMC6418428

[ref38] Bronesky D. Wu Z. Marzi S. Walter P. Geissmann T. Moreau K.. (2016). *Staphylococcus aureus* RNAIII and its regulon link quorum sensing, stress responses, metabolic adaptation, and regulation of virulence gene expression. Annu. Rev. Microbiol. 70, 299–316. 10.1146/annurev-micro-102215-095708 27482744

[ref39] Bronsard J. Pascreau G. Sassi M. Mauro T. Augagneur Y. Felden B. (2017). sRNA and cis-antisense sRNA identification in *Staphylococcus aureus* highlights an unusual sRNA gene cluster with one encoding a secreted peptide. Sci. Rep. 7:4565. 10.1038/s41598-017-04786-3, PMID: 28676719 PMC5496865

[ref40] Brouns S. J. J. Jore M. M. Lundgren M. Westra E. R. Slijkhuis R. J. H. Snijders A. P. L.. (2008). Small CRISPR RNAs guide antiviral defense in prokaryotes. Science 321, 960–964. 10.1126/science.1159689, PMID: 18703739 PMC5898235

[ref41] Brownlee G. G. (1971). Sequence of 6S RNA of *E. coli*. Nat. New Biol. 229, 147–149. 10.1038/newbio229147a0, PMID: 4929322

[ref42] Burenina O. Y. Oretskaya T. S. Kubareva E. A. (2017). Non-coding RNAs as transcriptional regulators in eukaryotes. Acta Nat. 9, 13–25. 10.32607/20758251-2017-9-4-13-25, PMID: 29340213 PMC5762824

[ref43] Calderón I. L. Morales E. H. Collao B. Calderón P. F. Chahuán C. A. Acuña L. G.. (2014). Role of *Salmonella* Typhimurium small RNAs RyhB-1 and RyhB-2 in the oxidative stress response. Res. Microbiol. 165, 30–40. 10.1016/j.resmic.2013.10.008, PMID: 24239962

[ref44] Carrier M.-C. Lalaouna D. Massé E. (2018). Broadening the definition of bacterial small RNAs: characteristics and mechanisms of action. Annu. Rev. Microbiol. 72, 141–161. 10.1146/annurev-micro-090817-062607, PMID: 30200848

[ref45] Catalan-Moreno A. Cela M. Menendez-Gil P. Irurzun N. Caballero C. J. Caldelari I.. (2021). RNA thermoswitches modulate *Staphylococcus aureus* adaptation to ambient temperatures. Nucleic Acids Res. 49, 3409–3426. 10.1093/nar/gkab117, PMID: 33660769 PMC8034633

[ref46] Cech T. R. Steitz J. A. (2014). The noncoding RNA revolution-trashing old rules to forge new ones. Cell 157, 77–94. 10.1016/j.cell.2014.03.008 24679528

[ref47] Chabelskaya S. Bordeau V. Felden B. (2014). Dual RNA regulatory control of a *Staphylococcus aureus* virulence factor. Nucleic Acids Res. 42, 4847–4858. 10.1093/nar/gku119, PMID: 24510101 PMC4005698

[ref48] Chabelskaya S. Gaillot O. Felden B. (2010). A *Staphylococcus aureus* small RNA is required for bacterial virulence and regulates the expression of an immune-evasion molecule. PLoS Pathog. 6:e1000927. 10.1371/journal.ppat.1000927, PMID: 20532214 PMC2880579

[ref49] Chao Y. Papenfort K. Reinhardt R. Sharma C. M. Vogel J. (2012). An atlas of Hfq-bound transcripts reveals 3’ UTRs as a genomic reservoir of regulatory small RNAs. EMBO J. 31, 4005–4019. 10.1038/emboj.2012.229, PMID: 22922465 PMC3474919

[ref50] Chao Y. Vogel J. (2016). A 3’ UTR-derived small RNA provides the regulatory noncoding arm of the inner membrane stress response. Mol. Cell 61, 352–363. 10.1016/j.molcel.2015.12.023, PMID: 26805574

[ref51] Chareyre S. Barras F. Mandin P. (2019). A small RNA controls bacterial sensitivity to gentamicin during iron starvation. PLoS Genet. 15:e1008078. 10.1371/journal.pgen.1008078, PMID: 31009454 PMC6497325

[ref52] Chareyre S. Mandin P. (2018). Bacterial iron homeostasis regulation by sRNAs. Microbiol. Spectr. 6. 10.1128/microbiolspec.RWR-0010-2017, PMID: 29573257 PMC11633579

[ref53] Chen L. Huang C. Wang X. Shan G. (2015). Circular RNAs in eukaryotic cells. Curr. Genomics 16, 312–318. 10.2174/1389202916666150707161554, PMID: 27047251 PMC4763969

[ref54] Chen J. Morita T. Gottesman S. (2019). Regulation of transcription termination of small RNAs and by small RNAs: molecular mechanisms and biological functions. Front. Cell. Infect. Microbiol. 9:201. 10.3389/fcimb.2019.00201, PMID: 31249814 PMC6582626

[ref55] Chevalier C. Boisset S. Romilly C. Masquida B. Fechter P. Geissmann T.. (2010). *Staphylococcus aureus* RNAIII binds to two distant regions of coa mRNA to arrest translation and promote mRNA degradation. PLoS Pathog. 6:e1000809. 10.1371/journal.ppat.1000809, PMID: 20300607 PMC2837412

[ref56] Christiansen J. K. Larsen M. H. Ingmer H. Søgaard-Andersen L. Kallipolitis B. H. (2004). The RNA-binding protein Hfq of *Listeria monocytogenes*: role in stress tolerance and virulence. J. Bacteriol. 186, 3355–3362. 10.1128/JB.186.11.3355-3362.2004, PMID: 15150220 PMC415768

[ref57] Christiansen J. K. Nielsen J. S. Ebersbach T. Valentin-Hansen P. Søgaard-Andersen L. Kallipolitis B. H. (2006). Identification of small Hfq-binding RNAs in *Listeria monocytogenes*. RNA 12, 1383–1396. 10.1261/rna.49706 16682563 PMC1484441

[ref58] Chunhua M. Yu L. Yaping G. Jie D. Qiang L. Xiaorong T.. (2012). The expression of LytM is down-regulated by RNAIII in *Staphylococcus aureus*. J. Basic Microbiol. 52, 636–641. 10.1002/jobm.201100426, PMID: 22581788

[ref59] Correia Santos S. Bischler T. Westermann A. J. Vogel J. (2021). MAPS integrates regulation of actin-targeting effector SteC into the virulence control network of *Salmonella* small RNA PinT. Cell Rep. 34:108722. 10.1016/j.celrep.2021.108722, PMID: 33535041

[ref60] Cotter R. I. McPhie P. Gratzer W. B. (1967). Internal organization of the ribosome. Nature 216, 864–868. 10.1038/216864a0, PMID: 6074964

[ref61] Crick F. H. (1958). On protein synthesis. Symp. Soc. Exp. Biol. 12, 138–163. PMID: 13580867

[ref62] Crick F. (1970). Central dogma of molecular biology. Nature 227, 561–563. 10.1038/227561a0, PMID: 4913914

[ref63] Cue D. Lei M. G. Lee C. Y. (2012). Genetic regulation of the intercellular adhesion locus in staphylococci. Front. Cell. Infect. Microbiol. 2:38. 10.3389/fcimb.2012.00038, PMID: 23061050 PMC3459252

[ref64] Dar D. Shamir M. Mellin J. R. Koutero M. Stern-Ginossar N. Cossart P.. (2016). Term-seq reveals abundant ribo-regulation of antibiotics resistance in bacteria. Science 352:aad9822. 10.1126/science.aad9822, PMID: 27120414 PMC5756622

[ref65] Dar D. Sorek R. (2018). Bacterial noncoding RNAs excised from within protein-coding transcripts. MBio 9, e01730–e01818. 10.1128/mBio.01730-18, PMID: 30254125 PMC6156199

[ref66] Das S. Lindemann C. Young B. C. Muller J. Osterreich B. Ternette N.. (2016). Natural mutations in a *Staphylococcus aureus* virulence regulator attenuate cytotoxicity but permit bacteremia and abscess formation. Proc. Natl. Acad. Sci. U. S. A. 113, E3101–E3110. 10.1073/pnas.1520255113, PMID: 27185949 PMC4896717

[ref67] Deana A. Belasco J. G. (2005). Lost in translation: the influence of ribosomes on bacterial mRNA decay. Genes Dev. 19, 2526–2533. 10.1101/gad.1348805, PMID: 16264189

[ref68] DebRoy S. Gebbie M. Ramesh A. Goodson J. R. Cruz M. R. van Hoof A.. (2014). Riboswitches. A riboswitch-containing sRNA controls gene expression by sequestration of a response regulator. Science 345, 937–940. 10.1126/science.1255091, PMID: 25146291 PMC4356242

[ref69] Denham E. L. (2020). The sponge RNAs of bacteria—how to find them and their role in regulating the post-transcriptional network. Biochim. Biophys Acta Gene Regul. Mech. 1863:194565. 10.1016/j.bbagrm.2020.194565, PMID: 32475775

[ref70] Derksen M. Mertens V. Pruijn G. J. M. (2015). RNase P-mediated sequence-specific cleavage of RNA by engineered external guide sequences. Biomol. Ther. 5, 3029–3050. 10.3390/biom5043029, PMID: 26569326 PMC4693268

[ref71] Dühring U. Axmann I. M. Hess W. R. Wilde A. (2006). An internal antisense RNA regulates expression of the photosynthesis gene isiA. Proc. Natl. Acad. Sci. U. S. A. 103, 7054–7058. 10.1073/pnas.0600927103 16636284 PMC1459017

[ref72] Dunman P. M. Murphy E. Haney S. Palacios D. Tucker-Kellogg G. Wu S.. (2001). Transcription profiling-based identification of *Staphylococcus aureus* genes regulated by the agr and/or sarA loci. J. Bacteriol. 183, 7341–7353. 10.1128/JB.183.24.7341-7353.2001, PMID: 11717293 PMC95583

[ref73] Dutta T. Srivastava S. (2018). Small RNA-mediated regulation in bacteria: a growing palette of diverse mechanisms. Gene 656, 60–72. 10.1016/j.gene.2018.02.068, PMID: 29501814

[ref74] Ellis T. N. Kuehn M. J. (2010). Virulence and immunomodulatory roles of bacterial outer membrane vesicles. Microbiol. Mol. Biol. Rev. 74, 81–94. 10.1128/MMBR.00031-09, PMID: 20197500 PMC2832350

[ref75] Eyraud A. Tattevin P. Chabelskaya S. Felden B. (2014). A small RNA controls a protein regulator involved in antibiotic resistance in *Staphylococcus aureus*. Nucleic Acids Res. 42, 4892–4905. 10.1093/nar/gku149, PMID: 24557948 PMC4005690

[ref76] Felden B. Cattoir V. (2018). Bacterial adaptation to antibiotics through regulatory RNAs. Antimicrob. Agents Chemother. 62, e02503–e02517. 10.1128/AAC.02503-17, PMID: 29530859 PMC5923175

[ref77] Feng L. Rutherford S. T. Papenfort K. Bagert J. D. van Kessel J. C. Tirrell D. A.. (2015). A qrr noncoding RNA deploys four different regulatory mechanisms to optimize quorum-sensing dynamics. Cell 160, 228–240. 10.1016/j.cell.2014.11.051, PMID: 25579683 PMC4313533

[ref78] Ferrara S. Falcone M. Macchi R. Bragonzi A. Girelli D. Cariani L.. (2017). The PAPI-1 pathogenicity island-encoded small RNA PesA influences *Pseudomonas aeruginosa* virulence and modulates pyocin S3 production. PLoS One 12:e0180386. 10.1371/journal.pone.0180386, PMID: 28665976 PMC5493400

[ref79] Figueroa-Bossi N. Valentini M. Malleret L. Fiorini F. Bossi L. (2009). Caught at its own game: regulatory small RNA inactivated by an inducible transcript mimicking its target. Genes Dev. 23, 2004–2015. 10.1101/gad.541609, PMID: 19638370 PMC2751969

[ref80] Foster T. J. (2005). Immune evasion by staphylococci. Nat. Rev. Microbiol. 3, 948–958. 10.1038/nrmicro1289, PMID: 16322743

[ref81] Fröhlich K. S. Papenfort K. Berger A. A. Vogel J. (2012). A conserved RpoS-dependent small RNA controls the synthesis of major porin OmpD. Nucleic Acids Res. 40, 3623–3640. 10.1093/nar/gkr1156 22180532 PMC3333887

[ref82] Fröhlich K. S. Papenfort K. Fekete A. Vogel J. (2013). A small RNA activates CFA synthase by isoform-specific mRNA stabilization. EMBO J. 32, 2963–2979. 10.1038/emboj.2013.222, PMID: 24141880 PMC3831309

[ref83] Gao W. Guérillot R. Lin Y. H. Tree J. Beaume M. François P.. (2020). Comparative transcriptomic and functional assessments of linezolid-responsive small RNA genes in *Staphylococcus aureus*. mSystems 5, e00665–e00719. 10.1128/mSystems.00665-19, PMID: 31911464 PMC6946794

[ref84] Geisinger E. Adhikari R. P. Jin R. Ross H. F. Novick R. P. (2006). Inhibition of rot translation by RNAIII, a key feature of agr function. Mol. Microbiol. 61, 1038–1048. 10.1111/j.1365-2958.2006.05292.x, PMID: 16879652

[ref85] Geissmann T. Chevalier C. Cros M. J. Boisset S. Fechter P. Noirot C.. (2009). A search for small noncoding RNAs in *Staphylococcus aureus* reveals a conserved sequence motif for regulation. Nucleic Acids Res. 37, 7239–7257. 10.1093/nar/gkp668, PMID: 19786493 PMC2790875

[ref86] Germain-Amiot N. Augagneur Y. Camberlein E. Nicolas I. Lecureur V. Rouillon A.. (2019). A novel *Staphylococcus aureus* cis-trans type I toxin-antitoxin module with dual effects on bacteria and host cells. Nucleic Acids Res. 47, 1759–1773. 10.1093/nar/gky1257 30544243 PMC6393315

[ref87] Giangrossi M. Prosseda G. Tran C. N. Brandi A. Colonna B. Falconi M. (2010). A novel antisense RNA regulates at transcriptional level the virulence gene icsA of *Shigella flexneri*. Nucleic Acids Res. 38, 3362–3375. 10.1093/nar/gkq025, PMID: 20129941 PMC2879508

[ref88] Gilbert W. (1986). Origin of life: the RNA world. Nature 319:618. 10.1038/319618a0

[ref89] Gimpel M. Brantl S. (2017). Dual-function small regulatory RNAs in bacteria. Mol. Microbiol. 103, 387–397. 10.1111/mmi.13558 27750368

[ref90] Gong H. Vu G.-P. Bai Y. Chan E. Wu R. Yang E.. (2011). A Salmonella small non-coding RNA facilitates bacterial invasion and intracellular replication by modulating the expression of virulence factors. PLoS Pathog. 7:e1002120. 10.1371/journal.ppat.1002120, PMID: 21949647 PMC3174252

[ref91] Göpel Y. Khan M. A. Görke B. (2014). Ménage à trois: post-transcriptional control of the key enzyme for cell envelope synthesis by a base-pairing small RNA, an RNase adaptor protein, and a small RNA mimic. RNA Biol. 11, 433–442. 10.4161/rna.28301, PMID: 24667238 PMC4152352

[ref92] Gorski S. A. Vogel J. Doudna J. A. (2017). RNA-based recognition and targeting: sowing the seeds of specificity. Nat. Rev. Mol. Cell Biol. 18, 215–228. 10.1038/nrm.2016.174, PMID: 28196981

[ref93] Gripenland J. Netterling S. Loh E. Tiensuu T. Toledo-Arana A. Johansson J. (2010). RNAs: regulators of bacterial virulence. Nat. Rev. Microbiol. 8, 857–866. 10.1038/nrmicro2457, PMID: 21079634

[ref94] Gruber C. C. Sperandio V. (2014). Posttranscriptional control of microbe-induced rearrangement of host cell actin. MBio 5, e01025–e01113. 10.1128/mBio.01025-13, PMID: 24425733 PMC3903284

[ref95] Gruber C. C. Sperandio V. (2015). Global analysis of posttranscriptional regulation by GlmY and GlmZ in enterohemorrhagic *Escherichia coli* O157:H7. Infect. Immun. 83, 1286–1295. 10.1128/IAI.02918-14, PMID: 25605763 PMC4363437

[ref96] Guerrier-Takada C. Gardiner K. Marsh T. Pace N. Altman S. (1983). The RNA moiety of ribonuclease P is the catalytic subunit of the enzyme. Cell 35, 849–857. 10.1016/0092-8674(83)90117-4, PMID: 6197186

[ref97] Gupta R. K. Luong T. T. Lee C. Y. (2015). RNAIII of the *Staphylococcus aureus* agr system activates global regulator MgrA by stabilizing mRNA. Proc. Natl. Acad. Sci. U. S. A. 112, 14036–14041. 10.1073/pnas.1509251112, PMID: 26504242 PMC4653210

[ref98] Hausmann S. Guimarães V. A. Garcin D. Baumann N. Linder P. Redder P. (2017). Both exo- and endo-nucleolytic activities of RNase J1 from *Staphylococcus aureus* are manganese dependent and active on triphosphorylated 5'-ends. RNA Biol. 14, 1431–1443. 10.1080/15476286.2017.1300223, PMID: 28277929 PMC5711453

[ref99] Heidrich N. Bauriedl S. Barquist L. Li L. Schoen C. Vogel J. (2017). The primary transcriptome of *Neisseria meningitidis* and its interaction with the RNA chaperone Hfq. Nucleic Acids Res. 45, 6147–6167. 10.1093/nar/gkx168, PMID: 28334889 PMC5449619

[ref100] Hindley J. (1967). Fractionation of 32P-labelled ribonucleic acids on polyacrylamide gels and their characterization by fingerprinting. J. Mol. Biol. 30, 125–136. 10.1016/0022-2836(67)90248-3, PMID: 4865141

[ref101] Hoagland M. B. Stephenson M. L. Scott J. F. Hecht L. I. Zamecnik P. C. (1958). A soluble ribonucleic acid intermediate in protein synthesis. J. Biol. Chem. 231, 241–257. 10.1016/S0021-9258(19)77302-5, PMID: 13538965

[ref102] Howden B. P. Beaume M. Harrison P. F. Hernandez D. Schrenzel J. Seemann T.. (2013). Analysis of the small RNA transcriptional response in multidrug-resistant *Staphylococcus aureus* after antimicrobial exposure. Antimicrob. Agents Chemother. 57, 3864–3874. 10.1128/AAC.00263-13, PMID: 23733475 PMC3719707

[ref103] Hoyos M. Huber M. Förstner K. U. Papenfort K. (2020). Gene autoregulation by 3’ UTR-derived bacterial small RNAs. elife 9:e58836. 10.7554/eLife.58836, PMID: 32744240 PMC7398697

[ref104] Huntzinger E. Boisset S. Saveanu C. Benito Y. Geissmann T. Namane A.. (2005). *Staphylococcus aureus* RNAIII and the endoribonuclease III coordinately regulate spa gene expression. EMBO J. 24, 824–835. 10.1038/sj.emboj.7600572 15678100 PMC549626

[ref105] Hussein H. Fris M. E. Salem A. H. Wiemels R. E. Bastock R. A. Righetti F.. (2019). An unconventional RNA-based thermosensor within the 5’ UTR of *Staphylococcus aureus* cidA. PLoS One 14:e0214521. 10.1371/journal.pone.0214521, PMID: 30933991 PMC6443170

[ref106] Hüttenhofer A. Noller H. F. (1994). Footprinting mRNA-ribosome complexes with chemical probes. EMBO J. 13, 3892–3901. 10.1002/j.1460-2075.1994.tb06700.x, PMID: 8070416 PMC395302

[ref107] Ikeda Y. Yagi M. Morita T. Aiba H. (2011). Hfq binding at RhlB-recognition region of RNase E is crucial for the rapid degradation of target mRNAs mediated by sRNAs in *Escherichia coli*. Mol. Microbiol. 79, 419–432. 10.1111/j.1365-2958.2010.07454.x, PMID: 21219461

[ref108] Ikemura T. Dahlberg J. E. (1973). Small ribonucleic acids of *Escherichia coli*. I. Characterization by polyacrylamide gel electrophoresis and fingerprint analysis. J. Biol. Chem. 248, 5024–5032. 10.1016/S0021-9258(19)43666-1, PMID: 4577761

[ref109] Ingavale S. van Wamel W. Luong T. T. Lee C. Y. Cheung A. L. (2005). Rat/MgrA, a regulator of autolysis, is a regulator of virulence genes in *Staphylococcus aureus*. Infect. Immun. 73, 1423–1431. 10.1128/IAI.73.3.1423-1431.2005, PMID: 15731040 PMC1064946

[ref110] Janssen K. H. Diaz M. R. Gode C. J. Wolfgang M. C. Yahr T. L. (2018). RsmV, a small noncoding regulatory RNA in *Pseudomonas aeruginosa* that sequesters RsmA and RsmF from target mRNAs. J. Bacteriol. 200, e00277–e00318. 10.1128/JB.00277-18, PMID: 29866805 PMC6060366

[ref111] Janssen B. D. Hayes C. S. (2012). The tmRNA ribosome-rescue system. Adv. Protein Chem. Struct. Biol. 86, 151–191. 10.1016/B978-0-12-386497-0.00005-0 22243584 PMC3358797

[ref112] Jia T. Liu B. Mu H. Qian C. Wang L. Li L.. (2021). A novel small RNA promotes motility and virulence of enterohemorrhagic *Escherichia coli* O157:H7 in response to ammonium. MBio 12, e03605–e03620. 10.1128/mBio.03605-20 33688013 PMC8092317

[ref113] Johansson J. Mandin P. Renzoni A. Chiaruttini C. Springer M. Cossart P. (2002). An RNA thermosensor controls expression of virulence genes in *Listeria monocytogenes*. Cell 110, 551–561. 10.1016/s0092-8674(02)00905-4 12230973

[ref114] Jørgensen M. G. Pettersen J. S. Kallipolitis B. H. (2020). sRNA-mediated control in bacteria: an increasing diversity of regulatory mechanisms. Biochim. Biophys Acta Gene Regul. Mech. 1863:194504. 10.1016/j.bbagrm.2020.194504, PMID: 32061884

[ref115] Joshi B. Singh B. Nadeem A. Askarian F. Wai S. N. Johannessen M.. (2020). Transcriptome profiling of *Staphylococcus aureus* associated extracellular vesicles reveals presence of small RNA-cargo. Front. Mol. Biosci. 7:566207. 10.3389/fmolb.2020.566207, PMID: 33521050 PMC7838569

[ref116] Kavita K. de Mets F. Gottesman S. (2018). New aspects of RNA-based regulation by Hfq and its partner sRNAs. Curr. Opin. Microbiol. 42, 53–61. 10.1016/j.mib.2017.10.014, PMID: 29125938 PMC10367044

[ref117] Kim J. N. (2016). Roles of two RyhB paralogs in the physiology of *Salmonella enterica*. Microbiol. Res. 186–187, 146–152. 10.1016/j.micres.2016.04.004 27242152

[ref118] Kim J. N. Kwon Y. M. (2013). Identification of target transcripts regulated by small RNA RyhB homologs in *Salmonella*: RyhB-2 regulates motility phenotype. Microbiol. Res. 168, 621–629. 10.1016/j.micres.2013.06.002, PMID: 23831078

[ref119] Kim S. Reyes D. Beaume M. Francois P. Cheung A. (2014). Contribution of teg49 small RNA in the 5' upstream transcriptional region of sarA to virulence in *Staphylococcus aureus*. Infect. Immun. 82, 4369–4379. 10.1128/IAI.02002-14 25092913 PMC4187880

[ref120] Kinoshita-Daitoku R. Kiga K. Miyakoshi M. Otsubo R. Ogura Y. Sanada T.. (2021). A bacterial small RNA regulates the adaptation of *helicobacter pylori* to the host environment. Nat. Commun. 12:2085. 10.1038/s41467-021-22317-7, PMID: 33837194 PMC8035401

[ref121] Koeppen K. Hampton T. H. Jarek M. Scharfe M. Gerber S. A. Mielcarz D. W.. (2016). A novel mechanism of host-pathogen interaction through sRNA in bacterial outer membrane vesicles. PLoS Pathog. 12:e1005672. 10.1371/journal.ppat.1005672, PMID: 27295279 PMC4905634

[ref122] Kole R. Krainer A. R. Altman S. (2012). RNA therapeutics: beyond RNA interference and antisense oligonucleotides. Nat. Rev. Drug Discov. 11, 125–140. 10.1038/nrd3625, PMID: 22262036 PMC4743652

[ref123] Kortmann J. Narberhaus F. (2012). Bacterial RNA thermometers: molecular zippers and switches. Nat. Rev. Microbiol. 10, 255–265. 10.1038/nrmicro2730, PMID: 22421878

[ref124] Lalaouna D. Baude J. Wu Z. Tomasini A. Chicher J. Marzi S.. (2019). RsaC sRNA modulates the oxidative stress response of *Staphylococcus aureus* during manganese starvation. Nucleic Acids Res. 47, 9871–9887. 10.1093/nar/gkz728, PMID: 31504767 PMC6765141

[ref125] Lalaouna D. Carrier M.-C. Semsey S. Brouard J.-S. Wang J. Wade J. T.. (2015). A 3' external transcribed spacer in a tRNA transcript acts as a sponge for small RNAs to prevent transcriptional noise. Mol. Cell 58, 393–405. 10.1016/j.molcel.2015.03.013, PMID: 25891076

[ref126] Lalaouna D. Prevost K. Eyraud A. Masse E. (2017). Identification of unknown RNA partners using MAPS. Methods 117, 28–34. 10.1016/j.ymeth.2016.11.011, PMID: 27876680

[ref127] Lasa I. Toledo-Arana A. Dobin A. Villanueva M. de los Mozos I. R. Vergara-Irigaray M. (2011). Genome-wide antisense transcription drives mRNA processing in bacteria. Proc. Natl. Acad. Sci. U. S. A. 108, 20172–20177. 10.1073/pnas.1113521108 22123973 PMC3250193

[ref130] Leclerc J.-M. Dozois C. M. Daigle F. (2013). Role of the *Salmonella enterica* serovar Typhi Fur regulator and small RNAs RfrA and RfrB in iron homeostasis and interaction with host cells. Microbiology 159, 591–602. 10.1099/mic.0.064329-0, PMID: 23306672

[ref131] Lee E.-J. Groisman E. A. (2010). An antisense RNA that governs the expression kinetics of a multifunctional virulence gene. Mol. Microbiol. 76, 1020–1033. 10.1111/j.1365-2958.2010.07161.x, PMID: 20398218 PMC2909850

[ref132] Lee J. Y. H. Monk I. R. Gonçalves da Silva A. Seemann T. Chua K. Y. L. Kearns A.. (2018). Global spread of three multidrug-resistant lineages of *Staphylococcus epidermidis*. Nat. Microbiol. 3, 1175–1185. 10.1038/s41564-018-0230-7, PMID: 30177740 PMC6660648

[ref133] Lejars M. Hajnsdorf E. (2020). The world of asRNAs in gram-negative and gram-positive bacteria. Biochim. Biophys Acta Gene Regul. Mech. 1863:194489. 10.1016/j.bbagrm.2020.194489, PMID: 31935527

[ref134] Lemon K. P. Higgins D. E. Kolter R. (2007). Flagellar motility is critical for *Listeria monocytogenes* biofilm formation. J. Bacteriol. 189, 4418–4424. 10.1128/JB.01967-06, PMID: 17416647 PMC1913361

[ref128] Le Pabic H. Germain-Amiot N. Bordeau V. Felden B. (2015). A bacterial regulatory RNA attenuates virulence, spread and human host cell phagocytosis. Nucleic Acids Res. 43, 9232–9248. 10.1093/nar/gkv783, PMID: 26240382 PMC4627067

[ref129] Le Scornet A. Redder P. (2019). Post-transcriptional control of virulence gene expression in *Staphylococcus aureus*. Biochim. Biophys Acta Gene Regul. Mech. 1862, 734–741. 10.1016/j.bbagrm.2018.04.004, PMID: 29705591

[ref135] Lioliou E. Romilly C. Romby P. Fechter P. (2010). RNA-mediated regulation in bacteria: from natural to artificial systems. New Biotechnol. 27, 222–235. 10.1016/j.nbt.2010.03.002 20211281

[ref136] Liu M. Y. Gui G. Wei B. Preston J. F. Oakford L. Yüksel U.. (1997). The RNA molecule CsrB binds to the global regulatory protein CsrA and antagonizes its activity in *Escherichia coli*. J. Biol. Chem. 272, 17502–17510. 10.1074/jbc.272.28.17502, PMID: 9211896

[ref137] Liu Y. Mu C. Ying X. Li W. Wu N. Dong J.. (2011). RNAIII activates map expression by forming an RNA-RNA complex in *Staphylococcus aureus*. FEBS Lett. 585, 899–905. 10.1016/j.febslet.2011.02.021, PMID: 21349272

[ref138] Loh E. Dussurget O. Gripenland J. Vaitkevicius K. Tiensuu T. Mandin P.. (2009). A trans-acting riboswitch controls expression of the virulence regulator PrfA in *Listeria monocytogenes*. Cell 139, 770–779. 10.1016/j.cell.2009.08.046, PMID: 19914169

[ref139] Loh E. Righetti F. Eichner H. Twittenhoff C. Narberhaus F. (2018). RNA thermometers in bacterial pathogens. Microbiol. Spectr. 6. 10.1128/microbiolspec.RWR-0012-2017, PMID: 29623874 PMC11633587

[ref140] Luirink J. Dobberstein B. (1994). Mammalian and *Escherichia coli* signal recognition particles. Mol. Microbiol. 11, 9–13. 10.1111/j.1365-2958.1994.tb00284.x 8145649

[ref141] Luong T. T. Dunman P. M. Murphy E. Projan S. J. Lee C. Y. (2006). Transcription profiling of the mgrA regulon in *Staphylococcus aureus*. J. Bacteriol. 188, 1899–1910. 10.1128/JB.188.5.1899-1910.2006, PMID: 16484201 PMC1426550

[ref142] Luz B. S. R. D. Nicolas A. Chabelskaya S. Rodovalho V. de Rezende Rodovalho V. Le Loir Y.. (2021). Environmental plasticity of the RNA content of *Staphylococcus aureus* extracellular vesicles. Front. Microbiol. 12:634226. 10.3389/fmicb.2021.634226, PMID: 33776967 PMC7990786

[ref143] Mader U. Nicolas P. Depke M. Pane-Farre J. Debarbouille M. van der Kooi-Pol M. M.. (2016). *Staphylococcus aureus* transcriptome architecture: from laboratory to infection-mimicking conditions. PLoS Genet. 12:e1005962. 10.1371/journal.pgen.1005962, PMID: 27035918 PMC4818034

[ref144] Mandin P. Repoila F. Vergassola M. Geissmann T. Cossart P. (2007). Identification of new noncoding RNAs in *Listeria monocytogenes* and prediction of mRNA targets. Nucleic Acids Res. 35, 962–974. 10.1093/nar/gkl1096, PMID: 17259222 PMC1807966

[ref145] Manna A. C. Kim S. Cengher L. Corvaglia A. Leo S. Francois P.. (2018). Small RNA teg49 is derived from a sarA transcript and regulates virulence genes independent of SarA in *Staphylococcus aureus*. Infect. Immun. 86, e00635–e00717. 10.1128/IAI.00635-17, PMID: 29133345 PMC5778362

[ref146] Massé E. Escorcia F. E. Gottesman S. (2003). Coupled degradation of a small regulatory RNA and its mRNA targets in *Escherichia coli*. Genes Dev. 17, 2374–2383. 10.1101/gad.1127103, PMID: 12975324 PMC218075

[ref147] Massé E. Gottesman S. (2002). A small RNA regulates the expression of genes involved in iron metabolism in *Escherichia coli*. Proc. Natl. Acad. Sci. U. S. A. 99, 4620–4625. 10.1073/pnas.032066599 11917098 PMC123697

[ref148] McDaniel B. A. M. Grundy F. J. Artsimovitch I. Henkin T. M. (2003). Transcription termination control of the S box system: direct measurement of S-adenosylmethionine by the leader RNA. Proc. Natl. Acad. Sci. U. S. A. 100, 3083–3088. 10.1073/pnas.0630422100 12626738 PMC152250

[ref149] Mediati D. G. Wu S. Wu W. Tree J. J. (2021). Networks of resistance: small RNA control of antibiotic resistance. Trends Genet. 37, 35–45. 10.1016/j.tig.2020.08.016 32951948

[ref150] Melamed S. Adams P. P. Zhang A. Zhang H. Storz G. (2020). RNA-RNA interactomes of ProQ and Hfq reveal overlapping and competing roles. Mol. Cell 77, 411.e7–425.e7. 10.1016/j.molcel.2019.10.022 31761494 PMC6980735

[ref151] Melamed S. Peer A. Faigenbaum-Romm R. Gatt Y. E. Reiss N. Bar A.. (2016). Global mapping of small RNA-target interactions in bacteria. Mol. Cell 63, 884–897. 10.1016/j.molcel.2016.07.026 27588604 PMC5145812

[ref152] Mellin J. R. Cossart P. (2012). The non-coding RNA world of the bacterial pathogen *Listeria monocytogenes*. RNA Biol. 9, 372–378. 10.4161/rna.19235 22336762

[ref153] Mellin J. R. Cossart P. (2015). Unexpected versatility in bacterial riboswitches. Trends Genet. 31, 150–156. 10.1016/j.tig.2015.01.005, PMID: 25708284

[ref154] Mellin J. R. Koutero M. Dar D. Nahori M.-A. Sorek R. Cossart P. (2014). Riboswitches. Sequestration of a two-component response regulator by a riboswitch-regulated noncoding RNA. Science 345, 940–943. 10.1126/science.1255083, PMID: 25146292

[ref155] Mellin J. R. Tiensuu T. Bécavin C. Gouin E. Johansson J. Cossart P. (2013). A riboswitch-regulated antisense RNA in *Listeria monocytogenes*. Proc. Natl. Acad. Sci. U. S. A. 110, 13132–13137. 10.1073/pnas.1304795110 23878253 PMC3740843

[ref156] Melson E. M. Kendall M. M. (2019). The sRNA DicF integrates oxygen sensing to enhance enterohemorrhagic *Escherichia coli* virulence via distinctive RNA control mechanisms. Proc. Natl. Acad. Sci. U. S. A. 116, 14210–14215. 10.1073/pnas.1902725116, PMID: 31235565 PMC6628830

[ref157] Menendez-Gil P. Toledo-Arana A. (2020). Bacterial 3’UTRs: a useful resource in post-transcriptional regulation. Front. Mol. Biosci. 7:617633. 10.3389/fmolb.2020.617633, PMID: 33490108 PMC7821165

[ref158] Mironov A. S. Gusarov I. Rafikov R. Lopez L. E. Shatalin K. Kreneva R. A.. (2002). Sensing small molecules by nascent RNA: a mechanism to control transcription in bacteria. Cell 111, 747–756. 10.1016/S0092-8674(02)01134-0, PMID: 12464185

[ref159] Miyakoshi M. Chao Y. Vogel J. (2015a). Cross talk between ABC transporter mRNAs via a target mRNA-derived sponge of the GcvB small RNA. EMBO J. 34, 1478–1492. 10.15252/embj.201490546 25630703 PMC4474525

[ref160] Miyakoshi M. Chao Y. Vogel J. (2015b). Regulatory small RNAs from the 3' regions of bacterial mRNAs. Curr. Opin. Microbiol. 24, 132–139. 10.1016/j.mib.2015.01.013 25677420

[ref161] Miyakoshi M. Matera G. Maki K. Sone Y. Vogel J. (2019). Functional expansion of a TCA cycle operon mRNA by a 3' end-derived small RNA. Nucleic Acids Res. 47, 2075–2088. 10.1093/nar/gky1243, PMID: 30541135 PMC6393394

[ref162] Mizuno T. Chou M. Y. Inouye M. (1984). A unique mechanism regulating gene expression: translational inhibition by a complementary RNA transcript (micRNA). Proc. Natl. Acad. Sci. U. S. A. 81, 1966–1970. 6201848 10.1073/pnas.81.7.1966 PMC345417

[ref163] Morfeldt E. Taylor D. von Gabain A. Arvidson S. (1995). Activation of alpha-toxin translation in *Staphylococcus aureus* by the trans-encoded antisense RNA, RNAIII. EMBO J. 14, 4569–4577. 10.1002/j.1460-2075.1995.tb00136.x, PMID: 7556100 PMC394549

[ref164] Moriano-Gutierrez S. Bongrand C. Essock-Burns T. Wu L. McFall-Ngai M. J. Ruby E. G. (2020). The noncoding small RNA SsrA is released by *Vibrio fischeri* and modulates critical host responses. PLoS Biol. 18:e3000934. 10.1371/journal.pbio.3000934, PMID: 33141816 PMC7665748

[ref165] Morita T. Maki K. Aiba H. (2005). RNase E-based ribonucleoprotein complexes: mechanical basis of mRNA destabilization mediated by bacterial noncoding RNAs. Genes Dev. 19, 2176–2186. 10.1101/gad.1330405, PMID: 16166379 PMC1221888

[ref166] Morrison J. M. Miller E. W. Benson M. A. Alonzo F. 3rd Yoong P. Torres V. J.. (2012). Characterization of SSR42, a novel virulence factor regulatory RNA that contributes to the pathogenesis of a *Staphylococcus aureus* USA300 representative. J. Bacteriol. 194, 2924–2938. 10.1128/JB.06708-11, PMID: 22493015 PMC3370614

[ref167] Mraheil M. A. Billion A. Mohamed W. Mukherjee K. Kuenne C. Pischimarov J.. (2011). The intracellular sRNA transcriptome of *Listeria monocytogenes* during growth in macrophages. Nucleic Acids Res. 39, 4235–4248. 10.1093/nar/gkr033, PMID: 21278422 PMC3105390

[ref168] Müller P. Gimpel M. Wildenhain T. Brantl S. (2019). A new role for CsrA: promotion of complex formation between an sRNA and its mRNA target in *Bacillus subtilis*. RNA Biol. 16, 972–987. 10.1080/15476286.2019.1605811, PMID: 31043113 PMC6546359

[ref169] Ng Kwan Lim E. Sasseville C. Carrier M.-C. Massé E. (2021). Keeping up with RNA-based regulation in bacteria: new roles for RNA binding proteins. Trends Genet. 37, 86–97. 10.1016/j.tig.2020.09.014, PMID: 33077249

[ref170] Nielsen J. S. Larsen M. H. Lillebæk E. M. S. Bergholz T. M. Christiansen M. H. G. Boor K. J.. (2011). A small RNA controls expression of the chitinase ChiA in *Listeria monocytogenes*. PLoS One 6:e19019. 10.1371/journal.pone.0019019, PMID: 21533114 PMC3078929

[ref171] Nielsen J. S. Lei L. K. Ebersbach T. Olsen A. S. Klitgaard J. K. Valentin-Hansen P.. (2010). Defining a role for Hfq in gram-positive bacteria: evidence for Hfq-dependent antisense regulation in *Listeria monocytogenes*. Nucleic Acids Res. 38, 907–919. 10.1093/nar/gkp1081 19942685 PMC2817478

[ref172] Nitzan M. Fechter P. Peer A. Altuvia Y. Bronesky D. Vandenesch F.. (2015). A defense-offense multi-layered regulatory switch in a pathogenic bacterium. Nucleic Acids Res. 43, 1357–1369. 10.1093/nar/gkv001, PMID: 25628364 PMC4330369

[ref173] Novick R. P. Iordanescu S. Projan S. J. Kornblum J. Edelman I. (1989). pT181 plasmid replication is regulated by a countertranscript-driven transcriptional attenuator. Cell 59, 395–404. 10.1016/0092-8674(89)90300-0, PMID: 2478296

[ref174] Novick R. P. Ross H. F. Projan S. J. Kornblum J. Kreiswirth B. Moghazeh S. (1993). Synthesis of staphylococcal virulence factors is controlled by a regulatory RNA molecule. EMBO J. 12, 3967–3975. 10.1002/j.1460-2075.1993.tb06074.x, PMID: 7691599 PMC413679

[ref175] Nudler E. (2006). Flipping riboswitches. Cell 126, 19–22. 10.1016/j.cell.2006.06.024, PMID: 16839869

[ref177] Obana N. Shirahama Y. Abe K. Nakamura K. (2010). Stabilization of *Clostridium perfringens* collagenase mRNA by VR-RNA-dependent cleavage in 5' leader sequence. Mol. Microbiol. 77, 1416–1428. 10.1111/j.1365-2958.2010.07258.x, PMID: 20572941

[ref176] O’Neil H. S. Marquis H. (2006). *Listeria monocytogenes* flagella are used for motility, not as adhesins, to increase host cell invasion. Infect. Immun. 74, 6675–6681. 10.1128/IAI.00886-06, PMID: 16982842 PMC1698079

[ref178] Oscarsson J. Tegmark-Wisell K. Arvidson S. (2006). Coordinated and differential control of aureolysin (aur) and serine protease (sspA) transcription in *Staphylococcus aureus* by sarA, rot and agr (RNAIII). Int. J. Med. Microbiol. 296, 365–380. 10.1016/j.ijmm.2006.02.019, PMID: 16782403

[ref179] Padalon-Brauch G. Hershberg R. Elgrably-Weiss M. Baruch K. Rosenshine I. Margalit H.. (2008). Small RNAs encoded within genetic islands of *Salmonella* Typhimurium show host-induced expression and role in virulence. Nucleic Acids Res. 36, 1913–1927. 10.1093/nar/gkn050, PMID: 18267966 PMC2330248

[ref180] Papenfort K. Bouvier M. Mika F. Sharma C. M. Vogel J. (2010). Evidence for an autonomous 5' target recognition domain in an Hfq-associated small RNA. Proc. Natl. Acad. Sci. U. S. A. 107, 20435–20440. 10.1073/pnas.1009784107 21059903 PMC2996696

[ref181] Papenfort K. Förstner K. U. Cong J.-P. Sharma C. M. Bassler B. L. (2015). Differential RNA-seq of *Vibrio cholerae* identifies the VqmR small RNA as a regulator of biofilm formation. Proc. Natl. Acad. Sci. U. S. A. 112, E766–E775. 10.1073/pnas.1500203112, PMID: 25646441 PMC4343088

[ref182] Papenfort K. Pfeiffer V. Lucchini S. Sonawane A. Hinton J. C. D. Vogel J. (2008). Systematic deletion of *Salmonella* small RNA genes identifies CyaR, a conserved CRP-dependent riboregulator of OmpX synthesis. Mol. Microbiol. 68, 890–906. 10.1111/j.1365-2958.2008.06189.x, PMID: 18399940

[ref183] Papenfort K. Pfeiffer V. Mika F. Lucchini S. Hinton J. C. Vogel J. (2006). SigmaE-dependent small RNAs of *Salmonella* respond to membrane stress by accelerating global omp mRNA decay. Mol. Microbiol. 62, 1674–1688. 10.1111/j.1365-2958.2006.05524.x, PMID: 17427289 PMC1804206

[ref184] Papenfort K. Sun Y. Miyakoshi M. Vanderpool C. K. Vogel J. (2013). Small RNA-mediated activation of sugar phosphatase mRNA regulates glucose homeostasis. Cell 153, 426–437. 10.1016/j.cell.2013.03.003 23582330 PMC4151517

[ref185] Papenfort K. Vanderpool C. K. (2015). Target activation by regulatory RNAs in bacteria. FEMS Microbiol. Rev. 39, 362–378. 10.1093/femsre/fuv016, PMID: 25934124 PMC4542691

[ref186] Peñaloza D. Acuña L. G. Barros M. J. Núñez P. Montt F. Gil F.. (2021). The small RNA RyhB homologs from *Salmonella* Typhimurium restrain the intracellular growth and modulate the SPI-1 gene expression within RAW264.7 macrophages. Microorganisms 9:635. 10.3390/microorganisms9030635, PMID: 33803635 PMC8002944

[ref187] Pennisi E. (2010). Shining a light on the genome’s “dark matter.” Science 330:1614. 10.1126/science.330.6011.1614, PMID: 21163986

[ref188] Pernitzsch S. R. Tirier S. M. Beier D. Sharma C. M. (2014). A variable homopolymeric G-repeat defines small RNA-mediated posttranscriptional regulation of a chemotaxis receptor in *Helicobacter pylori*. Proc. Natl. Acad. Sci. U. S. A. 111, E501–E510. 10.1073/pnas.1315152111 24474799 PMC3910625

[ref189] Pfeiffer V. Papenfort K. Lucchini S. Hinton J. C. D. Vogel J. (2009). Coding sequence targeting by MicC RNA reveals bacterial mRNA silencing downstream of translational initiation. Nat. Struct. Mol. Biol. 16, 840–846. 10.1038/nsmb.1631, PMID: 19620966

[ref190] Pichon C. Felden B. (2005). Small RNA genes expressed from *Staphylococcus aureus* genomic and pathogenicity islands with specific expression among pathogenic strains. Proc. Natl. Acad. Sci. U. S. A. 102, 14249–14254. 10.1073/pnas.0503838102 16183745 PMC1242290

[ref191] Pichon C. Felden B. (2007). Proteins that interact with bacterial small RNA regulators. FEMS Microbiol. Rev. 31, 614–625. 10.1111/j.1574-6976.2007.00079.x, PMID: 17655690

[ref192] Pinel-Marie M. L. Brielle R. Felden B. (2014). Dual toxic-peptide-coding *Staphylococcus aureus* RNA under antisense regulation targets host cells and bacterial rivals unequally. Cell Rep. 7, 424–435. 10.1016/j.celrep.2014.03.012, PMID: 24703849

[ref193] Pinel-Marie M.-L. Brielle R. Riffaud C. Germain-Amiot N. Polacek N. Felden B. (2021). RNA antitoxin SprF1 binds ribosomes to attenuate translation and promote persister cell formation in *Staphylococcus aureus*. Nat. Microbiol. 6, 209–220. 10.1038/s41564-020-00819-2, PMID: 33398097

[ref194] Pitman S. Cho K. H. (2015). The mechanisms of virulence regulation by small noncoding RNAs in low GC gram-positive pathogens. Int. J. Mol. Sci. 16, 29797–29814. 10.3390/ijms161226194, PMID: 26694351 PMC4691137

[ref195] Pourciau C. Lai Y.-J. Gorelik M. Babitzke P. Romeo T. (2020). Diverse mechanisms and circuitry for global regulation by the RNA-binding protein CsrA. Front. Microbiol. 11:601352. 10.3389/fmicb.2020.601352, PMID: 33193284 PMC7652899

[ref196] Quereda J. J. Cossart P. (2017). Regulating bacterial virulence with RNA. Annu. Rev. Microbiol. 71, 263–280. 10.1146/annurev-micro-030117-020335, PMID: 28886688

[ref197] Quereda J. J. García-Del Portillo F. Pucciarelli M. G. (2016). *Listeria monocytogenes* remodels the cell surface in the blood-stage. Environ. Microbiol. Rep. 8, 641–648. 10.1111/1758-2229.12416, PMID: 27085096

[ref198] Quereda J. J. Ortega A. D. Pucciarelli M. G. García-Del Portillo F. (2014). The *Listeria* small RNA Rli27 regulates a cell wall protein inside eukaryotic cells by targeting a long 5’-UTR variant. PLoS Genet. 10:e1004765. 10.1371/journal.pgen.1004765, PMID: 25356775 PMC4214639

[ref199] Raina M. King A. Bianco C. Vanderpool C. K. (2018). Dual-function RNAs. Microbiol. Spectr. 6:10. 10.1128/microbiolspec.RWR-0032-2018, PMID: 30191807 PMC6130917

[ref200] Redder P. (2018). Molecular and genetic interactions of the RNA degradation machineries in Firmicute bacteria. Wiley Interdiscip. Rev. RNA 9. 10.1002/wrna.1460, PMID: 29314657

[ref201] Rice K. C. Nelson J. B. Patton T. G. Yang S.-J. Bayles K. W. (2005). Acetic acid induces expression of the *Staphylococcus aureus* cidABC and lrgAB murein hydrolase regulator operons. J. Bacteriol. 187, 813–821. 10.1128/JB.187.3.813-821.2005, PMID: 15659658 PMC545714

[ref202] Rochat T. Bohn C. Morvan C. Le Lam T. N. Razvi F. Pain A.. (2018). The conserved regulatory RNA RsaE down-regulates the arginine degradation pathway in *Staphylococcus aureus*. Nucleic Acids Res. 46, 8803–8816. 10.1093/nar/gky584, PMID: 29986060 PMC6158497

[ref203] Rochat T. Delumeau O. Figueroa-Bossi N. Noirot P. Bossi L. Dervyn E.. (2015). Tracking the elusive function of *Bacillus subtilis* Hfq. PLoS One 10:e0124977. 10.1371/journal.pone.0124977, PMID: 25915524 PMC4410918

[ref204] Romeo T. (1998). Global regulation by the small RNA-binding protein CsrA and the non-coding RNA molecule CsrB. Mol. Microbiol. 29, 1321–1330. 10.1046/j.1365-2958.1998.01021.x 9781871

[ref205] Romilly C. Lays C. Tomasini A. Caldelari I. Benito Y. Hammann P.. (2014). A non-coding RNA promotes bacterial persistence and decreases virulence by regulating a regulator in *Staphylococcus aureus*. PLoS Pathog. 10:e1003979. 10.1371/journal.ppat.1003979, PMID: 24651379 PMC3961350

[ref206] Sáenz-Lahoya S. Bitarte N. García B. Burgui S. Vergara-Irigaray M. Valle J.. (2019). Noncontiguous operon is a genetic organization for coordinating bacterial gene expression. Proc. Natl. Acad. Sci. U. S. A. 116, 1733–1738. 10.1073/pnas.1812746116 30635413 PMC6358700

[ref207] Saïd-Salim B. Dunman P. M. McAleese F. M. Macapagal D. Murphy E. McNamara P. J.. (2003). Global regulation of *Staphylococcus aureus* genes by rot. J. Bacteriol. 185, 610–619. 10.1128/JB.185.2.610-619.2003, PMID: 12511508 PMC145333

[ref208] Salvail H. Caron M.-P. Bélanger J. Massé E. (2013). Antagonistic functions between the RNA chaperone Hfq and an sRNA regulate sensitivity to the antibiotic colicin. EMBO J. 32, 2764–2778. 10.1038/emboj.2013.205, PMID: 24065131 PMC3801439

[ref209] Sauder A. B. Kendall M. M. (2018). After the fact(or): posttranscriptional gene regulation in enterohemorrhagic *Escherichia coli* O157:H7. J. Bacteriol. 200, e00228–e00318. 10.1128/JB.00228-18, PMID: 29967119 PMC6148468

[ref210] Sayed N. Jousselin A. Felden B. (2012). A cis-antisense RNA acts in trans in *Staphylococcus aureus* to control translation of a human cytolytic peptide. Nat. Struct. Mol. Biol. 19, 105–112. 10.1038/nsmb.2193 22198463

[ref211] Schoenfelder S. M. K. Lange C. Prakash S. A. Marincola G. Lerch M. F. Wencker F. D. R.. (2019). The small non-coding RNA RsaE influences extracellular matrix composition in *Staphylococcus epidermidis* biofilm communities. PLoS Pathog. 15:e1007618. 10.1371/journal.ppat.1007618, PMID: 30870530 PMC6435200

[ref212] Sedlyarova N. Shamovsky I. Bharati B. K. Epshtein V. Chen J. Gottesman S.. (2016). sRNA-mediated control of transcription termination in *E. coli*. Cell 167, 111.e13–121.e13. 10.1016/j.cell.2016.09.004 27662085 PMC5040353

[ref213] Seidl K. Müller S. François P. Kriebitzsch C. Schrenzel J. Engelmann S.. (2009). Effect of a glucose impulse on the CcpA regulon in *Staphylococcus aureus*. BMC Microbiol. 9:95. 10.1186/1471-2180-9-95, PMID: 19450265 PMC2697999

[ref214] Serganov A. Yuan Y.-R. Pikovskaya O. Polonskaia A. Malinina L. Phan A. T.. (2004). Structural basis for discriminative regulation of gene expression by adenine- and guanine-sensing mRNAs. Chem. Biol. 11, 1729–1741. 10.1016/j.chembiol.2004.11.018, PMID: 15610857 PMC4692365

[ref215] Sesto N. Wurtzel O. Archambaud C. Sorek R. Cossart P. (2013). The excludon: a new concept in bacterial antisense RNA-mediated gene regulation. Nat. Rev. Microbiol. 11, 75–82. 10.1038/nrmicro2934, PMID: 23268228

[ref216] Sharma C. M. Darfeuille F. Plantinga T. H. Vogel J. (2007). A small RNA regulates multiple ABC transporter mRNAs by targeting C/A-rich elements inside and upstream of ribosome-binding sites. Genes Dev. 21, 2804–2817. 10.1101/gad.447207, PMID: 17974919 PMC2045133

[ref217] Sievers S. Lund A. Menendez-Gil P. Nielsen A. Storm Mollerup M. Lambert Nielsen S.. (2015). The multicopy sRNA LhrC controls expression of the oligopeptide-binding protein OppA in *Listeria monocytogenes*. RNA Biol. 12, 985–997. 10.1080/15476286.2015.1071011, PMID: 26176322 PMC4615310

[ref218] Sievers S. Sternkopf Lillebæk E. M. Jacobsen K. Lund A. Mollerup M. S. Nielsen P. K.. (2014). A multicopy sRNA of *Listeria monocytogenes* regulates expression of the virulence adhesin LapB. Nucleic Acids Res. 42, 9383–9398. 10.1093/nar/gku630 25034691 PMC4132741

[ref219] Singh R. Ray P. (2014). Quorum sensing-mediated regulation of staphylococcal virulence and antibiotic resistance. Future Microbiol. 9, 669–681. 10.2217/fmb.14.31, PMID: 24957093

[ref220] Smirnov A. Förstner K. U. Holmqvist E. Otto A. Günster R. Becher D.. (2016). Grad-seq guides the discovery of ProQ as a major small RNA-binding protein. Proc. Natl. Acad. Sci. U. S. A. 113, 11591–11596. 10.1073/pnas.1609981113, PMID: 27671629 PMC5068311

[ref221] Sonnleitner E. Pusic P. Wolfinger M. T. Bläsi U. (2020). Distinctive regulation of carbapenem susceptibility in *Pseudomonas aeruginosa* by Hfq. Front. Microbiol. 11:1001. 10.3389/fmicb.2020.01001, PMID: 32528439 PMC7264166

[ref222] Stork M. Di Lorenzo M. Welch T. J. Crosa J. H. (2007). Transcription termination within the iron transport-biosynthesis operon of Vibrio anguillarum requires an antisense RNA. J. Bacteriol. 189, 3479–3488. 10.1128/JB.00619-06, PMID: 17337574 PMC1855896

[ref223] Svensson S. L. Sharma C. M. (2016). Small RNAs in bacterial virulence and communication. Microbiol. Spectr. 4. 10.1128/microbiolspec.VMBF-0028-2015, PMID: 27337442

[ref224] Teixidó L. Cortés P. Bigas A. Alvarez G. Barbé J. Campoy S. (2010). Control by Fur of the nitrate respiration regulators NarP and NarL in *Salmonella enterica*. Int. Microbiol. 13, 33–39. 10.2436/20.1501.01.108, PMID: 20890837

[ref225] Thomason M. K. Storz G. (2010). Bacterial antisense RNAs: how many are there, and what are they doing? Annu. Rev. Genet. 44, 167–188. 10.1146/annurev-genet-102209-163523, PMID: 20707673 PMC3030471

[ref226] Thomason M. K. Voichek M. Dar D. Addis V. Fitzgerald D. Gottesman S.. (2019). A rhlI 5’ UTR-derived sRNA regulates RhlR-dependent quorum sensing in *Pseudomonas aeruginosa*. MBio 10, e02253–e02319. 10.1128/mBio.02253-19, PMID: 31594819 PMC6786874

[ref227] Tobe T. Yen H. Takahashi H. Kagayama Y. Ogasawara N. Oshima T. (2014). Antisense transcription regulates the expression of the enterohemorrhagic *Escherichia coli* virulence regulatory gene ler in response to the intracellular iron concentration. PLoS One 9:e101582. 10.1371/journal.pone.0101582 25006810 PMC4090186

[ref228] Toledo-Arana A. Dussurget O. Nikitas G. Sesto N. Guet-Revillet H. Balestrino D.. (2009). The *Listeria* transcriptional landscape from saprophytism to virulence. Nature 459, 950–956. 10.1038/nature08080, PMID: 19448609

[ref229] Toledo-Arana A. Lasa I. (2020). Advances in bacterial transcriptome understanding: from overlapping transcription to the excludon concept. Mol. Microbiol. 113, 593–602. 10.1111/mmi.14456 32185833 PMC7154746

[ref230] Toledo-Arana A. Repoila F. Cossart P. (2007). Small noncoding RNAs controlling pathogenesis. Curr. Opin. Microbiol. 10, 182–188. 10.1016/j.mib.2007.03.004, PMID: 17383223

[ref231] Tomizawa J. Itoh T. Selzer G. Som T. (1981). Inhibition of ColE1 RNA primer formation by a plasmid-specified small RNA. Proc. Natl. Acad. Sci. U. S. A. 78, 1421–1425. 10.1073/pnas.78.3.1421 6165011 PMC319142

[ref232] Tree J. J. Granneman S. McAteer S. P. Tollervey D. Gally D. L. (2014). Identification of bacteriophage-encoded anti-sRNAs in pathogenic *Escherichia coli*. Mol. Cell 55, 199–213. 10.1016/j.molcel.2014.05.006, PMID: 24910100 PMC4104026

[ref233] Updegrove T. B. Zhang A. Storz G. (2016). Hfq: the flexible RNA matchmaker. Curr. Opin. Microbiol. 30, 133–138. 10.1016/j.mib.2016.02.003, PMID: 26907610 PMC4821791

[ref234] Urban J. H. Vogel J. (2008). Two seemingly homologous noncoding RNAs act hierarchically to activate glmS mRNA translation. PLoS Biol. 6:e64. 10.1371/journal.pbio.0060064 18351803 PMC2267818

[ref235] Vakulskas C. A. Potts A. H. Babitzke P. Ahmer B. M. M. Romeo T. (2015). Regulation of bacterial virulence by Csr (Rsm) systems. Microbiol. Mol. Biol. Rev. 79, 193–224. 10.1128/MMBR.00052-14, PMID: 25833324 PMC4394879

[ref236] Vanderpool C. K. Gottesman S. (2004). Involvement of a novel transcriptional activator and small RNA in post-transcriptional regulation of the glucose phosphoenolpyruvate phosphotransferase system. Mol. Microbiol. 54, 1076–1089. 10.1111/j.1365-2958.2004.04348.x, PMID: 15522088

[ref237] Vannini A. Roncarati D. Danielli A. (2016). The cag-pathogenicity island encoded CncR1 sRNA oppositely modulates *Helicobacter pylori* motility and adhesion to host cells. Cell. Mol. Life Sci. 73, 3151–3168. 10.1007/s00018-016-2151-z, PMID: 26863876 PMC11108448

[ref238] Wagner E. G. H. Altuvia S. Romby P. (2002). Antisense RNAs in bacteria and their genetic elements. Adv. Genet. 46, 361–398. 10.1016/s0065-2660(02)46013-0 11931231

[ref239] Wagner E. G. Romby P. (2015). Small RNAs in bacteria and archaea: who they are, what they do, and how they do it. Adv. Genet. 90, 133–208. 10.1016/bs.adgen.2015.05.001 26296935

[ref240] Wang C. Chao Y. Matera G. Gao Q. Vogel J. (2020). The conserved 3’ UTR-derived small RNA NarS mediates mRNA crossregulation during nitrate respiration. Nucleic Acids Res. 48, 2126–2143. 10.1093/nar/gkz1168, PMID: 31863581 PMC7038943

[ref241] Wassarman K. M. Zhang A. Storz G. (1999). Small RNAs in *Escherichia coli*. Trends Microbiol. 7, 37–45. 10.1016/S0966-842X(98)01379-1, PMID: 10068996

[ref242] Waters L. S. Storz G. (2009). Regulatory RNAs in bacteria. Cell 136, 615–628. 10.1016/j.cell.2009.01.043, PMID: 19239884 PMC3132550

[ref243] Weilbacher T. Suzuki K. Dubey A. K. Wang X. Gudapaty S. Morozov I.. (2003). A novel sRNA component of the carbon storage regulatory system of *Escherichia coli*. Mol. Microbiol. 48, 657–670. 10.1046/j.1365-2958.2003.03459.x, PMID: 12694612

[ref244] Wen J. Fozo E. M. (2014). sRNA antitoxins: more than one way to repress a toxin. Toxins 6, 2310–2335. 10.3390/toxins6082310, PMID: 25093388 PMC4147584

[ref245] Westermann A. J. Förstner K. U. Amman F. Barquist L. Chao Y. Schulte L. N.. (2016). Dual RNA-seq unveils noncoding RNA functions in host-pathogen interactions. Nature 529, 496–501. 10.1038/nature16547, PMID: 26789254

[ref246] Winkler W. Nahvi A. Breaker R. R. (2002). Thiamine derivatives bind messenger RNAs directly to regulate bacterial gene expression. Nature 419, 952–956. 10.1038/nature01145, PMID: 12410317

[ref247] Wurtzel O. Sesto N. Mellin J. R. Karunker I. Edelheit S. Bécavin C.. (2012). Comparative transcriptomics of pathogenic and non-pathogenic *Listeria* species. Mol. Syst. Biol. 8:583. 10.1038/msb.2012.11, PMID: 22617957 PMC3377988

[ref248] Yang B. Feng L. Wang F. Wang L. (2015). Enterohemorrhagic *Escherichia coli* senses low biotin status in the large intestine for colonization and infection. Nat. Commun. 6:6592. 10.1038/ncomms7592, PMID: 25791315 PMC4382993

[ref249] Yu J. Schneiders T. (2012). Tigecycline challenge triggers sRNA production in *Salmonella enterica* serovar Typhimurium. BMC Microbiol. 12:195. 10.1186/1471-2180-12-195, PMID: 22958399 PMC3511261

[ref250] Zhang Y. F. Han K. Chandler C. E. Tjaden B. Ernst R. K. Lory S. (2017). Probing the sRNA regulatory landscape of *P. aeruginosa*: post-transcriptional control of determinants of pathogenicity and antibiotic susceptibility. Mol. Microbiol. 106, 919–937. 10.1111/mmi.13857, PMID: 28976035 PMC5738928

[ref251] Zhang H. Zhang Y. Song Z. Li R. Ruan H. Liu Q.. (2020). sncRNAs packaged by *Helicobacter pylori* outer membrane vesicles attenuate IL-8 secretion in human cells. Int. J. Med. Microbiol. 310:151356. 10.1016/j.ijmm.2019.151356 31585715

[ref252] Zhao X. Zhang Y. Huang X. (2018). Pathogenicity-island-encoded regulatory RNAs regulate bacterial virulence and pathogenesis. Microb. Pathog. 125, 196–204. 10.1016/j.micpath.2018.09.028, PMID: 30227229

[ref253] Zhukova A. Fernandes L. G. Hugon P. Pappas C. J. Sismeiro O. Coppée J.-Y.. (2017). Genome-wide transcriptional start site mapping and sRNA identification in the pathogen *Leptospira interrogans*. Front. Cell. Infect. Microbiol. 7:10. 10.3389/fcimb.2017.00010, PMID: 28154810 PMC5243855

